# Comprehensive review of targeted therapy for colorectal cancer

**DOI:** 10.1038/s41392-020-0116-z

**Published:** 2020-03-20

**Authors:** Yuan-Hong Xie, Ying-Xuan Chen, Jing-Yuan Fang

**Affiliations:** Division of Gastroenterology and Hepatology, Shanghai Institute of Digestive Disease, State Key Laboratory for Oncogenes and Related Genes, Key Laboratory of Gastroenterology & Hepatology, Ministry of Health, Renji Hospital, School of Medicine, Shanghai Jiao Tong University, 145 Middle Shandong Road, 200001 Shanghai, China

**Keywords:** Gastrointestinal cancer, Cancer therapy

## Abstract

Colorectal cancer (CRC) is among the most lethal and prevalent malignancies in the world and was responsible for nearly 881,000 cancer-related deaths in 2018. Surgery and chemotherapy have long been the first choices for cancer patients. However, the prognosis of CRC has never been satisfying, especially for patients with metastatic lesions. Targeted therapy is a new optional approach that has successfully prolonged overall survival for CRC patients. Following successes with the anti-EGFR (epidermal growth factor receptor) agent cetuximab and the anti-angiogenesis agent bevacizumab, new agents blocking different critical pathways as well as immune checkpoints are emerging at an unprecedented rate. Guidelines worldwide are currently updating the recommended targeted drugs on the basis of the increasing number of high-quality clinical trials. This review provides an overview of existing CRC-targeted agents and their underlying mechanisms, as well as a discussion of their limitations and future trends.

## Introduction

### Current treatment for colorectal cancer

Colorectal cancer (CRC) ranks as the second most lethal cancer and the third most prevalent malignant tumor worldwide. In 2018, 1.8 million new CRC cases arose, and 881,000 deaths were reported, which accounted for nearly 10% of new cancer cases and deaths worldwide,^1^ and the number of new cases may increase to nearly 2.5 million in 2035.^2^ According to statistics in the USA, the death rate declined by ~50% in 2016 (13.7 per 10,000 patients) compared with that in 1970 (29.2 per 10,000 patients) because of the rapid development of screening methods and improved treatment methods. However, this trend seems to be observed only in highly developed countries.^2^ Meanwhile, the 5-year survival rate for CRC is ~64%, but drops to 12% for metastatic CRC, and further investigation is still required to develop effective approaches for medical intervention.^3^

Given the advances in primary and adjuvant treatments, the survival time in CRC has been improving. Typically, the ideal CRC treatment is to achieve complete removal of the tumor and metastases, which mostly requires surgical intervention.^4^ However, despite the emergence of numerous screening programs to reduce CRC incidence, nearly a quarter of CRCs are diagnosed at an advanced stage with metastases, and 20% of the remaining cases may develop metachronous metastases, which result in difficulties in curative surgical control and subsequent tumor-related deaths.^5–8^ For those patients with unresectable lesions or who are intolerant to surgery, the goal is maximum shrinkage of the tumor and suppression of further tumor spread and growth, and radiotherapy and chemotherapy are the leading strategies for controlling disease in such patients. Of note, in some cases, chemotherapy or radiotherapy might be applied before or after surgery as neoadjuvant or adjuvant treatment to maximally reduce and stabilize the tumor.^9–12^

### Chemotherapy

Current chemotherapy includes both single-agent therapy, which is mainly fluoropyrimidine (5-FU)-based, and multiple-agent regimens containing one or several drugs, including oxaliplatin (OX), irinotecan (IRI), and capecitabine (CAP or XELODA or XEL). Although studies have argued that first-line single-agent therapy is not inferior to combined regimens in terms of overall survival (OS),^13,14^ the combined therapy regimens FOLFOX (5-FU+OX), FOXFIRI (5-FU+IRI), XELOX or CAPOX (CAP+OX), and CAPIRI (CAP+OX) remain the mainstream approaches in first-line treatment, while patients with poor performance or at low risk of deterioration are recommended to receive single-agent therapy. When choosing additive agents, efficacy appears to be similar, and only adverse events may differ among different regimens.^12,15–17^ Emerging evidence does not support stronger efficacy in the multiple-agent regimen FOLOXIRI (5-FU+OX+IRI), which is infrequently applied because of its potential increased toxicity.^18,19^ Nonetheless, data from research performed in recent decades show that using chemotherapy in patients with CRC, especially those with metastases, has pushed their OS time to almost 20 months, resulting in chemotherapy becoming the backbone of CRC treatment.^15,20,21^ However, chemotherapy is associated with certain limitations, such as existing systemic toxicity, unsatisfying response rate, unpredictable innate and acquired resistance, and low tumor-specific selectivity. Therefore, massive investments have been pledged to develop new approaches to refine or even replace existing CRC chemotherapy.

### Targeted therapy

The idea of molecular targeted therapy has a relatively long history. The concept of a chemical that specifically targets a microorganism was first proposed in the early 1900s and expanded to cancer treatment in 1988,^22^ and this concept was renewed and has flourished in the past 20 years.^23^

Targeted therapies can work on cancerous cells by directly inhibiting cell proliferation, differentiation, and migration. The tumor microenvironment, including local blood vessels and immune cells, might also be altered by targeted drugs to impede tumor growth and enact stronger immune surveillance and attack. Small molecules, such as monoclonal antibodies, are major players in targeted therapies.^24–26^ Small molecules are a group of molecules with a molecular weight <900 Da that might penetrate into cells, mostly working within cells to inactivate selected enzymes, thereby interfering with tumor cell growth and even triggering apoptosis. Cyclin-dependent kinases, proteasomes, and poly ADP-ribose polymerase make up most of the molecular targets. Carfilzomib for multiple myeloma, ribociclib for metastatic breast cancer, and rucaparib for BRCA-positive ovarian cancer are a few examples.^23^ For targets outside cells, such as cell surface receptors or membrane-bound sites, monoclonal antibodies or therapeutic antibodies can recognize and bind them to directly regulate downstream cell cycle progression and cell death. In addition, certain monoclonal antibodies work on cells other than cancer cells, such as immune cells, which helps to manipulate the immune system to attack human cancer.

### Landscape of current CRC-targeted therapy

**Fig. 1:**
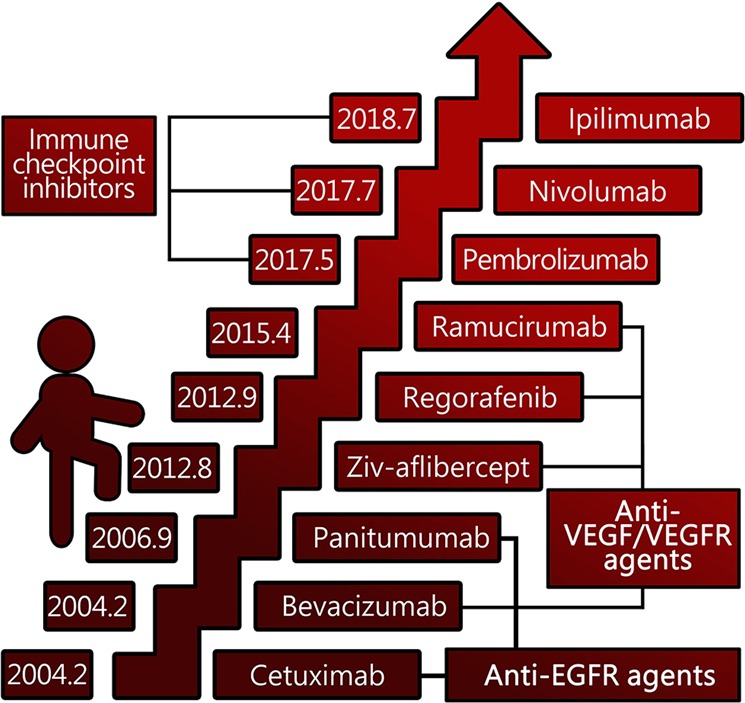
United States of America Food and Drug Administration (FDA)-approved targeted agents in colorectal cancer. VEGF: vascular endothelial growth factor; VEGFR: vascular endothelial growth factor receptor; EGFR: epidermal growth factor receptor

**Fig. 2:**
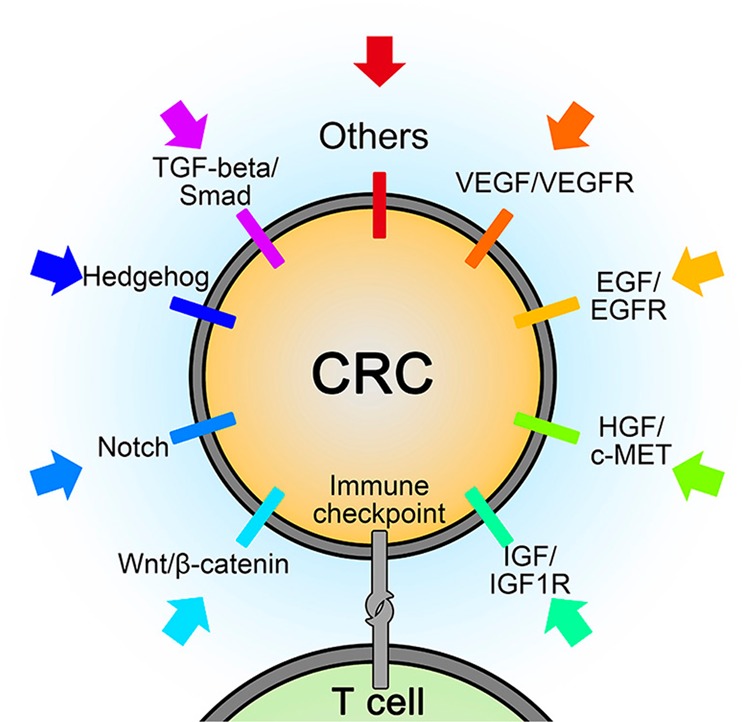
Pathways offering potential sites for targeted therapy. CRC: colorectal cancer; VEGF/VEGFR: vascular endothelial growth factor/vascular endothelial growth factor receptor; EGF/EGFR: epidermal growth factor/epidermal growth factor receptor; HGF: hepatocyte growth factor; c-MET: mesenchymal–epithelial transition factor; IGF/IGF-1R: insulin-like growth factor/ insulin-like growth factor 1 receptor; TGF: transforming growth factor

The first targeted agent for CRC approved by the Food and Drug Administration (FDA) was cetuximab in 2004, followed by bevacizumab in the same year, and emerging FDA-approved targeted drugs for CRC have been brought to market successively since then, with more on the way (Fig. 1). Numerous agents have been developed and brought into preclinical and clinical trials. The list of recommended CRC-targeted agents from guidelines such as those from the National Comprehensive Cancer Network (NCCN) is being updated quickly, given the unprecedented speed of the emergence of large trials (Fig. 2).

Various pathways mediating the initiation, progression, and migration of CRC, such as Wnt/β-catenin, Notch, Hedgehog, and TGF-β (transforming growth factor-β)/SMAD, as well as those capable of activating signaling cascades, such as phosphatidylinositol 3-kinase (PI3K)/AKT or RAS/rapidly accelerated fibrosarcoma (RAF), contain ideal sites for targeted therapy (Fig. 2).^27,28^ Given the complex downstream signaling and difficulties in completely inhibiting specific biological interactions, not all existing CRC-related pathways can be successfully interfered with, and current data cover only a few pathways in which experimentally identified targeted agents can be proved to be efficient in clinical studies, and a large group of targeted drugs remain in preclinical status or in phase I trials.

## The EGFR-related pathway

### Activities of the pathway

EGFR (epidermal growth factor receptor) belongs to the ErbB (erythroblastosis oncogene B)/HER (human epidermal growth factor receptor) family, which consists of four members: ErbB1 (EGFR/HER1), ErbB2 (Neu/HER2), ErbB3 (HER3), and ErbB4 (HER4).^29,30^ The ErbB receptors were first considered to be related to carcinogenesis almost 30 years ago and are quite unique among the multitude of receptor tyrosine kinases. The impaired kinase activity of HER3/ErbB3 and the absence of a direct HER2/ErbB2 ligand mean that these transmembrane glycoprotein can only be activated after homo- or heterodimerization with HER2, HER3, or HER4 through specific binding, mainly by EGF or TGF-α. Once activated, various downstream intracellular signaling pathways, including the RAS/RAF/MEK/ERK, PI3K/AKT, and JAK/STAT3 (Janus kinase/signal transducer and activator of transcription 3) pathways, are triggered to regulate cell growth, survival, and migration.^31–33^

Aberrant expression levels of EGFR and HER have been identified in a group of cancers, including glioma; melanoma; medulloblastoma; gastrointestinal tumors such as esophageal, colorectal, and gastric cancers; and cancers in the lung, breast, bladder, prostate, pancreas, and ovary.^29^ Overexpression of EGFR has been observed in 15–30% of breast cancers, 60% of NSCLCs (non-small-cell lung cancer), and 25–77% of CRCs, which might also indicate poor prognosis.^34–36^ HER2 overexpression occurs in ~20–30% of breast and ovarian cancers,^33,37^ in 3.8–36.6% of gastric cancers^38^ and in 1.3–47.7% of CRCs.^39^ HER3 showed higher expression in 83% of gastrointestinal tumors and 20% of breast, ovarian, and bladder cancers than in normal tissues;^33,40^ however, it was prohibited from becoming a drug target given difficulties in finding its ligand. HER4 remains controversial because both cancer-promoting and cancer-suppressing effects have been found.^41,42^ Therefore, substantial efforts are being made to develop mainstream targeted drugs for HER1 and HER2 while facing potential drug resistance caused by mutations of HER1 and HER2. For instance, mutations of EGFR and HER2 were found in 15–30% of NSCLC samples^43,44^ and in 1.6% of HER2-positive breast cancer cases.^45^

The typical ErbB receptor consists of a ligand-binding domain outside the cell, a transmembrane domain, and an intracellular domain with distinct tyrosine residues in the C-terminal region where subsequent phosphorylation may take place upon activation.^46^

Activation of EGFR triggers various downstream signaling pathways that mediate cellular proliferation or metabolism, playing vital roles in cancer initiation and progression. Activated EGFR initiates plasma recruitment of SOSs (son of sevenless homologs) to achieve RAS-RAF activation, which leads to phosphorylation of mitogen-activated protein kinase (MAPK or MEK) and activation of extracellular signal-related kinase (ERK), which might then translocate inside the nucleus to regulate the expression of transcription factors such as c-FOS and ELK1.^47–50^ It is worth mentioning that during RAS-RAF activation, the serine/threonine protein kinase in the RAF family, BRAF (BRAF proto-oncogene, serine/threonine kinase), plays vital roles in the RAS/RAF/MEK pathway. In contrast to RAS mutations, BRAF mutations, mostly comprising the V600E alteration, were found in 5–10% of metastatic CRC cases and cause activation of downstream MAPK regardless of RAS status.^51–54^

Activation of PI3K by RAS or direct activation by EGFR transforms the second messenger phosphatidylinositol-bisphosphate into phosphatidylinositol-trisphosphate (PIP3) through phosphorylation. PIP3 interacts with the SH3 domain of serine/threonine kinase PKB (also called AKT) recruited to the cell membrane. AKT plays significant roles in cell growth and apoptosis and works as a vital mediator in the ErbB-related pathway.^55–57^ In addition, the ErbB2-3 heterodimer is the strongest activator of the PI3K/AKT pathway among all the ErbB-dimer family members. Cancer and diabetes are closely related to poorly regulated AKT activity.^58,59^ AKT regulates cell cycle entry and survival via phosphorylation of forkhead box O, BCL2-associated agonist of cell death, and glycogen synthase kinase 3 (GSK-3), thereby preventing cellular apoptosis through mammalian target of rapamycin activation.^57–59^

Another vital protein that influences various cellular biological functions, such as cell motility, growth, differentiation, and membrane ruffle formation, which are mainly activated by EGFR, is phospholipase C-γ1 (PLC-γ1).^60–62^ This 145 kDa protein has one SH3 domain, two pleckstrin homology domains, and two SH2 domains that might interact with EGFR, eventually increasing enzyme activity to produce inositol-triphosphate (IP3) and diacylglycerol (DAG) from hydrolysis of phosphatidylinositol-bisphosphate.^63–67^ IP3 and DAG promote the release of intracellular Ca^2+^ and activate protein kinase C to promote carcinogenesis.^68,69^ Moreover, recent studies stated that the SH3 domain of PLC-γ1 might be of great importance in the interaction with EGFR. EGF mediates PLC-γ1 binding to AKT, altering its activity through the SH3 domain.^70^ In addition, PI3K enhancer, a nuclear GTPase that activates nuclear PI3K activity,^71^ dynamin-1, and Racl, which might enhance EGF-induced cell proliferation and migration, are regulated by the SH3 domain of PLC-γ1, acting as guanine nucleotide exchange factors.^70,72^

EGFR might directly bind to and phosphorylate STATs to enable them to dimerize and transfer into the nucleus, where they mediate cell growth, differentiation, and apoptosis by regulating related gene transcription.^73,74^ In addition, a nonreceptor tyrosine kinase (c-Src) acts on EGFR in an indirect way to govern STATs, which exerts a crucial effect. Overexpression of c-Src and EGFR occurs in many cancer cases, suggesting a close interaction between them and their potential contributions to tumor proliferation. The Src family is a group of nonreceptor tyrosine kinases that overlap with the STAT and PI3K pathways. SRCs enhance EGFR signaling through c-SRC-dependent phosphorylation and c-SRC-EGFR complex formation.^75,76^

### Targeting EGFR and EGFR-related pathways

Methods to target the EGFR pathway typically comprise anti-EGFR monoclonal antibodies and tyrosine kinase inhibitors (TKIs) aimed at intracellular kinases (Tables 1 and 2).

**Table 1:** Agents targeting EGFR and EGFR-related pathways in colorectal cancer

Agent	Key trial (NCT number)	Design (*N*)	Subject	Treatment	Main results		
					RR	OS	PFS
EGFR inhibitor							
Cetuximab	Crystal^80^	Phase III	mCRC	FOLFIRI + CETU	46.9%	19.9 m	8.9 m
	NCT00154102	(*N* = 364)			(OR 1.4*)	(HR 0.93)	(HR 0.85*)
				FOLFIRI	38.7%	18.6 m	8.0 m
Cetuximab	Tailor^98^	Phase III	mCRC (RAS-WT)^a^ First line	FOLFOX + CETU	61.1%	20.7 m	9.2 m
	NCT01228734	(*N* = 393)			(OR 2.41**)	(HR 0.76*)	(HR 0.69**)
				FOLFOX	39.5%	17.8 m	7.4 m
Panitumumab	PRIME^89,90^	Phase III	mCRC (KRAS-WT)	FOLFOX + PAN	57%	23.9 m	10.0 m
	NCT00364013	(*N* = 656)			(OR 1.47*)	(HR 0.88)	(HR 0.8*)
				FOLFOX	48%	19.7 m	8.6 m
Cetuximab	EPIC^94^	Phase III	mCRC	Cetuximab + Irinotecan	16.4%	10.7 m	4.0 m
		(*N* = 1298)	Second line			(HR 0.975)	(HR 0.692***)
				Irinotecan	4.2%	10 m	2.6 m
Panitumumab	PICCOLO^96^	Phase III	mCRC (KRAS-WT)	Panitumumab + Irinotecan	34%	10.4 m	NR
	ISRCTN	(*N* = 460)			(OR 4.12***)	(HR 1.01)	(HR 0.78*)
	93248876		Second line	Irinotecan	12%	10.5 m	NR
Panitumumab	20050181^508^	Phase III	mCRC (KRAS-WT)	FOLFIRI + Panitumumab	35%	14.5 m	5.9 m
		(*N* = 597)				(HR 0.85)	(HR 0.73**)
			Second line	FOLFIRI	10%	12.5 m	3.9 m
BRAF inhibitor with MEK inhibitor							
Vemurafenib	SWOG S1406^111^	Phase II	mCRC	Irinotecan + Cetuximab + Vemurafenib	16%	4.4 m	NR
	NCT02164916	(*N* = 106)	BRAF^V600^ mutated				
			Extended RAS-WT			(HR 0.42***)	
			Treatment refractory	Irinotecan + Cetuximab	4%	2.0 m	NR
Dabrafenib and trametinib (MEK inhibitor)	Corcoran^109^	Phase I/II	mCRC	Dabrafenib + Trametinib	12%	NR	3.5 m
		(*N* = 43)	BRAF^v600^ mutated				
Encorafenib + binimetinib (MEK inhibitor)	BEACON^113^	Phase III (safety lead-in results)	mCRC	ENCO + BINI + CETU	26%***	9 m	4.3 m
	NCT02928224		BRAF^V600E^ mutated			(HR=0.52***)	(HR 0.40***)
			Second or third line	ENCO+CETU	20%***	8.4 m	4.2 m
		(*N* = 30)		Irinotecan/FOLFIRI + CETU		(HR=0.60***)	(HR=0.38***)
					2%	5.4 m	1.5 m
HER2 inhibitor							
Trastuzumab + pertuzumab	MyPathway^124^	Phase IIa	mCRC	Trastuzumab + pertuzumab	32%	11.5 m	2.9 m
	NCT02091141	(*N* = 57)	HER2 amplified				
Trastuzumab + lapatinib	HERACLES^125^	Phase II	mCRC	Trastuzumab+lapatinib	30%	46 weeks	21 weeks
		(*N* = 27)	HER2 positive				
			KRAS exon 2 WT				
			Treatment refractory				

*mCRC* metastatic colorectal cancer, *RR* response rate, *OS* overall survival, *PFS* progression-free survival, *CETU* cetuximab, *PAN* panitumumab, *ENCO* encorafenib, *BINI* binimetinib, *NR* not reported, *WT* wild type, *m* months.

*<0.05; **<0.01; ***<0.001

**Table 2:** Agents targeting EGFR or EGFR-related pathway under clinical investigation

Name or ID	Targets	Condition	Phase	NCT identifier
PX-866	PI3K	mCRC	Phase 1/2	NCT01252628
		Advanced solid tumors	Phase 1	NCT00726583
Alpelisib	PI3K	CRC	Phase 1/2	NCT01719380
BKM120	PI3K	Previously treated CRC	Phase 1	NCT01304602
		RAS-wild-type CRC	Phase 1/2	NCT01591421
		Advanced solid tumors	Phase 1	NCT01068483
		Advanced solid tumors	Phase 1	NCT01571024
		PIK3CA-mutated cancers	Phase 2	NCT01501604
		Advanced solid tumors	Phase 1	NCT01576666
PF-05212384 Gedatolisib	PI3K/mTOR	KRAS/NRAS-wild-type mCRC	Phase 2	NCT01925274
		mCRC	Phase 1/2	NCT01937715
BEZ-235 Dactolisib	PI3K/mTOR	Advanced or metastatic solid tumors	Phase 1	NCT01195376
			Phase 1	NCT01337765
			Phase 1	NCT01285466
BGT-226	PI3K/mTOR	Advanced solid malignancies	Phase 1/2	NCT00600275
Temsirolimus CCI-770	mTOR	KRAS-mutated mCRC	Phase 2	NCT00827684
		Refractory CRC	Phase 1	NCT00593060
Everolimus RAD001	mTOR	mCRC	Phase 2	NCT01387880
		Refractory mCRC	Phase 1	NCT01154335
		Advanced CRC	Phase 1/2	NCT01139138
		mCRC	Phase 1/2	NCT01058655
		CRC	Phase 1/2	NCT01047293
Nab-rapamycin	mTOR	First-line CRC	Phase 1/2	NCT03439462
Ridaforolimus	mTOR	Malignant neoplasms	Phase 1	NCT01243762
MK-2206	AKT	CRC	Phase 2	NCT01802320
		CRC	Phase 2	NCT01333475
Enzastaurin	AKT	mCRC	Phase 2	NCT00612586
		mCRC	Phase 2	NCT00437268
		mCRC	Phase 2	NCT00192114
Napabucasin	STAT3	mCRC	Phase 2	NCT03647839
		Previously treated mCRC	Phase 3	NCT03522649
AZD-9150	STAT3	Multiple tumors including CRC	Phase 2	NCT02983578
TTI-101	STAT3	Multiple tumors including CRC	Phase 1	NCT03195699
Niclosamide	STAT3	CRC	Phase 1	NCT02687009
Pimasertib	MAPK	Solid tumors	Phase 1	NCT01390818
Cobimetinib	MAPK	Gastrointestinal and other tumors	Phase 1	NCT02876224
		Solid tumors	Phase 2	NCT02457793
		mCRC	Phase 3	NCT02788279
Refametinib	MEK	Advanced cancers	Phase 1	NCT01392521
TAK-733	MEK	Advanced malignancies	Phase 1	NCT00948467
RO4987655	MEK	Advanced solid tumors	Phase 1	NCT00817518
Selumetinib	MEK	Advanced solid tumors	Phase 1	NCT02586987
		Refractory solid tumors	Phase 1	NCT01217450
		Solid tumors and CRC	Phase 1	NCT01287130
		mCRC	Phase 2	NCT00514761
		Previously treated CRC	Phase 2	NCT01116271
		Advanced CRC	Phase 2	NCT01333475
PD-0325901	MEK	Solid tumors	Phase 1/2	NCT03905148
Neratinib	EGFR/HER2/4	RAS-mutated solid tumors	Phase 1	NCT03919292
		KRAS/NRAS/BRAF/PIK3CA-wild-type mCRC	Phase 2	NCT03457896
AZD-8931	EGFR/HER2/3	mCRC	Phase 2	NCT01862003
MEHD7945A	EGFR/HER3	KRAS-mutated cancers	Phase 1	NCT01986166
		KRAS-mutated mCRC	Phase 2	NCT01652482
Duligotuzumab	EGFR/HER3	KRAS-mutated cancers	Phase 1	NCT01986166
Erlotinib	EGFR	mCRC	Phase 3	NCT00264824
		mCRC	Phase 3	NCT00598156
		First-line mCRC	Phase 3	NCT01229813
Sym-004	EGFR	Advanced solid tumors	Phase 1/2	NCT01117428
		mCRC	Phase 2	NCT02083653
Gefitinib	EGFR	Refractory CRC	Phase 1/2	NCT00242788
		CRC in the second-line chemotherapy setting	Phase 2/3	NCT00234429
MM151	EGFR	Advanced solid tumors	Phase 1	NCT01520389
Afatinib	EGFR	Refractory mCRC	Phase 2	NCT01919879
		mCRC	Phase 2	NCT01152437
		Advanced CRC	Phase 2	NCT00801294
		Solid tumors	Phase 2	NCT02465060
MCLA-158	EGFR/Lgr5	Multiple tumors including CRC	Phase 1	NCT03526835
Seribantumab	HER3	Advanced cancer	Phase 1	NCT01451632
Dasatinib	Src	mCRC	Phase 2	NCT00504153
		Advanced or mCRC	Phase 1	NCT04164069
Saracatinib AZD0530	Src	Solid tumors	Phase 1	NCT00771979
GSK2118436	BRAF	mCRC	Phase 2	NCT03668431
		mCRC	Phase 2	NCT03428126
BMS-908662	BRAF	K-RAS/BRAF-mutated CRC	Phase 1/2	NCT01086267

*CRC* colorectal cancer, *mCRC* metastatic colorectal cancer, *PI3K* phosphoinositide 3-kinase, *AKT* protein kinase B, also known as PKB, *mTOR* mammalian target of rapamycin, *MEK* mitogen-activated protein kinase, *EGFR* epidermal growth factor receptor, *HER2/3/4* human epidermal growth factor 2/3/4, *MAPK* mitogen-activated protein kinase, *STAT3* signal transducer and activator of transcription 3

#### Cetuximab and panitumumab

In 1995, the first monoclonal antibody targeted to EGFR with convincing preclinical data was announced. Named cetuximab, it is a chimeric immunoglobulin G (IgG) antibody that induces EGFR internalization and degradation once bound to the external domain of EGFR.^77^ Cetuximab showed great potential in progression-free survival (PFS) improvement in patients with low response to single-agent IRI therapy, according to the BOND trial, which contributed to the FDA approval of cetuximab for metastatic CRC in 2004.^78^ Moreover, a subsequent study also confirmed that cetuximab treatment prolonged OS and PFS in patients with CRCs when previous treatment with fluoropyrimidine, IRI and OX failed or was contraindicated.^79^ Combinations of cetuximab with other existing chemotherapies also displayed promising results. The phase III CRYSTAL trial found that cetuximab plus the FOLFIRI regimen had better progression control (8.9 vs. 8 months, hazard ratio (HR) 0.85; *p* = 0.048) than FOLFIRI alone, although the OS was not significantly different (HR, 0.93; *p* = 0.31).^80^ Interestingly, in different studies investigating cetuximab combined with FOLFOX in metastatic patients with CRC,^81–83^ no significant PFS or OS improvement was identified given that the doses in FOLFOX might have differed between studies because of the impact of the crossover design, but this lack of improvements in PFS and OS has also now been ascribed to CRC molecular heterogeneity.^84^ Maintaining cetuximab alone after a FOLFOX plus cetuximab regimen was not inferior to maintaining combination therapy in terms of PFS, with fewer adverse reactions noted.^85^ Escalating to the maximal dose of cetuximab based on the intensity of skin rash in the EVEREST trial suggested that an overall response might be achieved but without OS improvement.^86^

Murine-human chimeric antibodies might cause immunogenic reactions; therefore, the fully humanized antibody panitumumab has been developed, which does not trigger antibody-dependent cell-mediated cytotoxicity like cetuximab does^87^ and showed a lower risk of hypersensitivity reactions (0.6–3.0% for panitumumab and 3.5–7.5% for cetuximab).^88^ The efficacy of panitumumab against CRC was evaluated in the PRIME trial when FOLFOX plus panitumumab was compared with FOLFOX alone, and the combination regimen achieved a better PFS (10 vs. 8.6 months, HR 0.80, *p* = 0.01) and OS than FOLFOX alone (23.9 vs. 19.7 months, HR = 0.88, *p* = 0.17), with further demonstrated significance in the updated survival analysis (HR = 0.83, *p* = 0.003) in patients with metastatic CRC.^89,90^

Maintenance with panitumumab and 5-FU/LV after panitumumab plus FOLFOX showed numerical improvement in PFS and OS compared with single-agent panitumumab in the retrospective analysis of the PRIME and PEAK trials.^91^ The toxicity of this combination did not increase, which was confirmed in the VALENTINO trial, in which maintaining single-agent panitumumab appeared to have shorter PFS (HR = 1.55, *p* = 0.011) than treatment with panitumumab combined with 5-FU/LV.^92^

Cetuximab and panitumumab are both FDA-approved agents for the first-line treatment of CRC. No inferiority or superiority was identified in the phase III ASPECCT study between these two drugs. Cetuximab resulted in an OS of 10.0 months, and the OS was 10.4 months for panitumumab (HR 0.97, *p* < 0.0007 for noninferiority), in which no obvious adverse events were noted other than the incidence of grade 3 or 4 hypomagnesemia (3% for cetuximab and 7% for panitumumab).^93^ This also indicated that antibody-dependent cell-mediated cytotoxicity was not a major mechanism for these agents. However, in terms of quality-adjusted life-years, panitumumab seemed to be more economically efficient than cetuximab.^93^

For second-line treatment of CRC or beyond, anti-EGFR agents might be low priority because in several studies cetuximab and panitumumab have been demonstrated to fail to reach statistically better PFS or OS for patients with CRC.^94–96^ In fact, only one study^97^ reported that panitumumab significantly prolonged PFS (8 vs. 7.3 weeks, HR = 0.54, *p* < 0.001) compared with best supportive care in patients with chemorefractory CRC with an acceptable rate of adverse events. In general, anti-EGFR agents are among the least attractive choices in second-line treatment, especially compared with anti-vascular endothelial growth factor (VEGF) agents, which will be discussed in a subsequent section.

Notably, subgroup analysis has indicated that both of these anti-EGFR agents are robustly beneficial to those patients with RAS-wild-type tumors in the CRYSTAL, PRIME, and TAILOR trials,^80,90,98^ even though negative outcomes were experienced in patients with RAS mutations (KRAS and NRAS exon 2, 3, and 4 mutations). Interestingly, left-sided CRC tends to be more enriched for EGFR expression than right-sided CRC, in which MSI or BRAF mutations are predominantly activated.^99^ This sidedness leads to different clinical outcomes, such that worse OS and PFS have been observed in right-sided CRC than in left-sided CRC regardless of the choice of chemotherapy regimen or targeted agent.^100,101^ This biological factor has also been validated in anti-EGFR agent trials: in terms of RR, PFS, and OS within RAS-wild-type patients, those with left-sided tumors were expected to have better clinical outcomes than those with right-sided cancers.^80,90,98^ As demonstrated above, BRAF mutations are independent from RAS mutations and are closely related to a low anti-EGFR response, and both the NCCN and ESMO guidelines recommend using cetuximab and panitumumab in confirmed BRAF-wild-type and RAS-wild-type patients.^102^

#### BRAF inhibitors

A higher incidence of mutated BRAF is found in melanoma than in CRC. The efficiency of BRAF inhibitors in BRAF-V600E-mutated melanoma prompted the development of a similar approach in CRC. A few studies investigated blocking BRAF or BRAF/MEK using vemurafenib or dabrafenib or using selective BRAF inhibitors and trametinib; however, a selective MEK inhibitor failed to improve the PFS or OS of patients with metastatic CRC, even though downstream MAPK activity was inhibited after drug administration. Some scholars have suggested that BRAF/MEK blockade might trigger feedback reactivation of EGFR, which would bypass activating MAPK via RAS.^103–105^ Preclinical research indicated that a combination of BRAF inhibitors and an upstream-pathway inhibitor might be superior to BRAF inhibition alone in terms of tumor growth control in BRAF-mutated CRC xenograft models.^104,106,107^ Subsequent studies focused on the combined use of BRAF inhibitors and EGFR inhibitors.^108,109^ Promising survival outcomes and response rates were observed in trials using vemurafenib combined with IRI and cetuximab for patients with BRAF-mutant CRC.^110,111^ In a phase II trial using encorafenib (a BRAF inhibitor) plus cetuximab, with or without alpelisib (ALP), the PFS and OS were improved compared with those seen in historical data.^112^ A triplet regimen consisting of dabrafenib, trametinib, and panitumumab achieved a better response rate than the doublet regimens (21% vs. 10% for dabrafenib + panitumumab or 0% for trametinib + panitumumab) in patients with BRAF-V600E-mutated CRCs.^109^ Similar results were reported for the ongoing BEACON trial, in which a triplet regimen of encorafenib, binimetinib (a MEK inhibitor), and cetuximab was well tolerated and exceeded previous efficacy outcomes for BRAF inhibitors.^113^ New evidence emerged suggesting that a triple regimen of encorafenib, binimetinib, and cetuximab offered significantly better survival benefit for patients with BRAF-mutated metastatic CRC than that achieved historically with a comparable rate of adverse events (OS: 9 vs. 5.4 months, HR = 0.52, *p* < 0.001; RR: 26% vs. 2%, *p* < 0.01).^113^ Second- or third-line regimens treating BRAF-V600E-mutated mCRC now may include anti-EGFR agents combined with vemurafenib + IRI or dabrafenib + trametinib or encorafenib + binimitinib, as recommended by the NCCN.

#### HER2 inhibitor

As discussed above, HER2 acts similarly to EGFR because it shares many downstream pathways, such as RAS/RAF/MEK and PI3K/AKT, and overexpression of HER provides one explanation for anti-EGFR resistance.^114–116^ Unlike the rate in breast cancer or gastric cancer, the rate of HER2 overexpression is relatively low (2–3%) and is independent of RAS or RAF mutation in patients with CRC.^117–119^ Preclinical studies revealed that HER2 amplification might compensate for EGFR blockade, and combined targeting of HER2 and EGFR inhibited tumor cell proliferation, producing an effect that was stronger than that achieved using either single agent alone.^115,118,120^ Several clinical trials have been developed to determine whether targeted agents against HER2-positive CRC (determined by immunohistochemistry (IHC), fluorescence in situ hybridization (FISH), or chromogenic in situ hybridization) can be as effective as those against breast cancer or gastric cancer. A few of these studies using a single HER2-targeted agent, with or without chemotherapy, were terminated early because of a low patient response rate or insignificant patient survival benefits.^121,122^ By contrast, dual-targeted HER2 therapy was found to be promising in preclinical research.^118,123^ In the phase II MyPathway trial, doublet treatment with trastuzumab, a classic HER2 inhibitor, and pertuzumab, a HER2 dimerization inhibitor, both of which are FDA approved in HER-positive breast cancer treatment, helped patients with HER2-amplified metastatic CRC to gain an overall response rate of 32%, a PFS of 2.9 months and an OS of 11.5 months, which may be even better in patients with RAS-wild-type CRCs (PFS: 5.3 months and OS: 14 months OS).^124^ Another dual anti-HER2 agent combination of trastuzumab and lapatinib (a TKI targeting both EGFR and HER2) against metastatic CRC was studied in the phase II HERACLES trial and reached an overall response rate of 30%, a PFS of 21 weeks and an OS of 46 weeks.^125^ In addition, this combination was capable of overcoming resistance to pertuzumab and trastuzumab doublet treatment.^126^ Given the low rate of HER2 overexpression and difficulties in identifying suitable dual-HER2 regimens, the HERACLES trial took great pains to find a potentially effective doublet regimen consisting of lapatinib;^125^ thus, targeting HER2 might act as a backup regimen for patients with RAS-wild-type HER2-positive CRC. Notably, left-sided colon tumors tend to overexpress HER2 more than those on the right side. Thus, anti-HER2 therapy might offer a new choice for anti-EGFR-resistant CRC.^125^

### EGFR resistance

Accumulating evidence shows that even patients with RAS-wild-type CRC might not benefit from EGFR-targeted therapy, which suggests that identifying certain factors predicting low anti-EGFR therapy response and introducing other agents or strategies to overcome resistance would be beneficial. Some of these factors are innate or intrinsic, some are acquired after anti-EGFR treatment, and some may occur in both situations.

#### RAS mutations

RAS mutations are found in nearly half of patients with CRC, most of whom also harbor KRAS or NRAS mutations (36% for KRAS and 3% for NRAS).^127^ However, data showed that not all KRAS-mutated patients developed EGFR resistance: 85–90% of patients had mutations in KRAS codons 12 and 13 (exon 2), which largely indicate EGFR therapy resistance.^128–130^ For other sites, such as KRAS G13D, the connection with drug resistance is uncertain.^131,132^ Moreover, even patients with wild-type KRAS exon 2 might have other RAS mutations in sites such as KRAS exons 3 and 4 and NRAS exons 2, 3, and 4, which are related to negative benefits from cetuximab or panitumumab treatment.^84,133^

#### PI3K mutations and PTEN loss

PI3K (encoding phosphatidylinositol-4,5-bisphosphate 3-kinase) mutations occur mostly in exons 9 and 20; mutations in exon 9 or exon 20 are found in 10–18% of patients with metastatic CRC and lead to constitutive activation of the downstream pathway to reverse EGFR-blocking effects in patients with CRC (response rate of 0% vs. 36.8% in mutated vs. nonmutated patients).^134,135^ PTEN (phosphatase and tensin homolog) is a suppressor in the PI3K/AKT pathway, the loss of which resulted in long-term tumor growth via activated PI3K/AKT and was found in 20–40% of patients with metastatic CRC.^136^ Theoretically, PTEN loss might be associated with EGFR blockade resistance; however, data from clinical studies remain contradictory.^137,138^ Given the low occurrence rate of these mutations in CRC, large trials are required for better confirmation.

#### EGFR alterations

Mutations in EGFR or low expression of EGFR or AREG (amphiregulin)/EREG (epiregulin), key ligands in the EGFR-specific autocrine loop, cause loss of target for anti-EGFR therapy, representing one of the major ways by which EGFR resistance develops in NSCLC and CRC. Although high EGFR levels might correlate slightly with stronger efficacy of anti-EGFR therapy, patients with low EGFR gene expression may benefit less from EGFR blockers than patients with high EGFR gene expression.^139,140^ Clinical studies also found that low AREG/EREG levels identified a low cetuximab response rate and vice versa.^141,142^ EGFR mutated sites vary, and the uncommon ones are linked to worse prognosis.^136^ For the common mutations, the T790M mutation is considered to be a primary alteration inducing EGFR TKI resistance, which is frequently observed in patients with NSCLC.^143^ For patients with CRCs, the EGFR S492R mutation in the extracellular domain of EGFR may be found in those receiving cetuximab and was responsible for their low drug response; however, they may still respond to panitumumab.^144^ New agents are being developed to maximize the affinity for mutated EGFR, such as Sym044^145,146^ and MM151,^147^ which might simultaneously target several different sites of the EGFR extracellular domain to overcome resistance to cetuximab or panitumumab, and both Sym044 and MM151 are in preclinical studies and clinical trials.

Compensative activation of alternative pathways, such as IGF-1R (insulin-like growth factor 1 receptor), JAK/STAT, c-MET, VEGF, and HER2, is responsible for acquired anti-EGFR resistance. Similar to EGFR, IGF-1R is bound by IGF1 or 2 and may activate RAS/RAF and PI3K/AKT signaling. Increased IGF-1R activation was noted in patients with CRC receiving cetuximab and was associated with a significantly lower response rate than that seen in patients without IGF-1R activation^148^ (22% vs. 65%, *p* = 0.002). This effect has also been observed in patients with breast cancer;^149^ thus, introducing an IGF-1R inhibitor combined with an EGFR blocker might be a practical solution. A phase III trial, combining the IGF-1R inhibitor dalotuzumab with cetuximab, showed numerically superior PFS and OS improvement in patients with CRC with IGF-1R-positive tumors,^150^ although preclinical studies did not support noticeable benefits from anti-IGF-1R treatment,^151^ which implied that more steps are needed for IGF-1R targeting. Persistent JAK/STAT activation might also be vital for EGFR-targeted resistance, although the increased level of STAT3 phosphorylation seen in in vivo and in vitro studies was related to gefitinib resistance, which could be overcome by silencing STAT3 in CRC cells.^152,153^

#### Bypass amplification and activation

c-MET and VEGF amplification and activation are discussed in the following parts of this review.

Another technique to develop novel anti-EGFR agents is to enclose conventional EGFR blockers within other agents such as nanoparticles, liposomes, and other protein-based drug delivery systems, which have shown promising tumor affinity and drug efficacy in several preclinical studies.^154^

## The VEGF/VEFGR pathway

### About the pathway

Angiogenesis, a physiological process by which new vessels form or reform from existing vessels, plays a vital role in tumor initiation, growth, and metastasis. Angiogenesis is also under complex regulation involving various proangiogenic and antiangiogenic factors, such as VEGF, fibroblast growth factors (FGFs), TGF-α, TGF-β, platelet-derived endothelial cell growth factor (PDGF), and angiopoietins produced from cancer or stromal cells.^155–157^ The relationship between neo-vessels and carcinogenesis remained theoretical until the identification of VEGF-A (also known as VEGF) and the production of its monoclonal antibody inhibitor, which finally demonstrated the tumor-promoting effect of angiogenesis.^158^ The VEGF family consists of five members (VEGF-A, -B, -C, and -D and placental growth factor (PIGF)), which may bind to endothelial cells via tyrosine kinase VEGF receptors. Vascular endothelial growth factor receptors (VEGFRs) are divided into three types, VEGFR-1, -2, and -3, along with the non-tyrosine kinase coreceptors neuropilin-1 (NP-1) and NP-2. The VEGF family may also interact with other proteins, such as integrins,^157,159–162^ to regulate angiogenesis, for example, by guiding the migration of endothelial cells.^163^ Among the complicated and diverse interactions between VEGF and VEGFR, VEGF-A, VEGF-B, and PIGF contribute predominantly to angiogenesis, while VEGF-C and VEGF-D tend to regulate lymphangiogenesis. VEGF-A and VEGF-B mainly bind to VEGFR-1 and VEGFR-2, which are mostly expressed on vascular endothelial cells and on some nonendothelial cells.^164^ VEGFR-3 is bound by VEGF-C and VEGF-D with greatest affinity and is expressed on endothelial lymphatic cells.^165^

VEGFR-1 is a 180 kDa member of the receptor tyrosine kinase family expressed on many kinds of cells, including epithelial cells, inflammatory cells, and cancer cells. VEGFR-1 has high affinity for VEGF-1 and relatively low affinity for VEGF-2 and PIGF. Interestingly, VEGFR-1 seems to make little contribution to cell proliferation during vascular formation. Instead, it regulates cell differentiation and migration, especially for epithelial cells,^163,166^ and promotes differentiation of epithelial cells during early vascular construction.^166^ In addition, activation of VEGFR-1 under pathological conditions in inflammatory cells mediates the activation of several downstream pathways, such as PI3K/AKT/MAPK/ERK, leading to upregulation of inflammatory cytokine production (TNF-α and some interleukins (IL-1β, IL-6, and IL-8)) and inflammatory cell migration. The detailed function of VEGFR-1 is not fully understood; however, it is believed to be a regulatory factor in angiogenesis. VEGFR-1 favors VEGF-A over VEGFR-2, and the interaction of PIGF with VEGFR-1 might allow VEGF-A to bind to VEGFR-2. Therefore, VEGFR-1 works as a decoy regulator to control the amount of free VEGF-A available to activate VEGFR-2 when angiogenic effects appear to be mediated by VEGF-A/VEGFR-2.^164,167,168^

In contrast to VEGFR-1, VEGFR-2 is actively involved in vascular formation. It has a molecular mass of 200–230 kDa and is mostly expressed on blood and lymphatic epithelial cells.^166^ VEGFR-2 mainly interacts with VEGF-A, and activated VEGFR-2 leads to phosphorylation of tyrosine residues and activation of various pathways, including the PLCγ and RAS/RAF/ERK/MAPK pathways, by which epithelial cell growth is promoted, and the PI3K/AKT pathway, by which cell apoptosis may be avoided.^156,157,161,163,166^ Moreover, adhesion molecules such as cadherins and β-catenin, which are activated by the PI3K and MAPK pathways, may further interact with VEGFR-2, causing deterioration of intercellular junction stability and epithelial cell cytoskeleton reorganization, thus elevating vascular permeability. Vascular permeability is also enhanced by epithelial cell production of endothelial nitric oxide synthase (eNOS) and nitric oxide (NO) via AKT protein kinase activation.^169^ The above observations indicate the proangiogenic effect of VEGFR-2 in physiological and pathological conditions. Activated VEGFR-2 contributes to the differentiation, proliferation, migration, and apoptosis resistance of epithelial cells, thereby increasing vascular tubulogenesis and permeability, which is very important for cancer angiogenesis and progression.

VEGFR-3, activated by VEGF-C and VEGF-D, contributes relatively independently to lymphatic vessel formation.^170,171^ Activated VEGFR-3 mediates the differentiation, migration, proliferation and survival of lymphatic endothelial cells by activating the RAS/MAPK/ERK pathway and the PI3K–AKT/PKB pathway.^169–171^ Although the VEGFR-3 expression level in tumor cells remains controversial, high levels of VEGF-C and VEGF-D have been observed in tumors with lymphatic metastasis, which is considered a potential explanation for cancer migration through lymphatic vessels.^172^

VEGF levels and VEGFR activity are elevated in patients with CRC and other cancers and are considered to be related to poor prognosis.^173–176^ Some tumor cells produce VEGF and express VEGFR, suggesting that VEGF works as both an autocrine factor and an endocrine factor in this situation. Increased VEGF levels were observed in very early stages of colorectal neoplasia, e.g., adenoma, and were even higher in later stages of cancer, especially in the metastatic stage.^177,178^ VEGF regulation is complex in CRC. Mutated K-RAS and p53, expression of COX-2, and hypoxia inducible factor 1 (HIF-1) induced by hypoxia from high tumor cell density might all contribute to VEGF-VEGFR activity alteration, resulting in cancer growth and migration.^178–181^ The proangiogenic effects of VEGF-VEGFR are important both in local sites supporting tumor progression and migration and in metastatic sites for neovascularization to support cancer survival and growth; therefore, anti-VEGF/VEGFR therapy might be developed to target both steps in tumor metastasis.

### Targeting angiogenesis

#### Bevacizumab: the milestone

The landmark trials based on antiangiogenic therapy for CRC were initiated in 2004, comprising the phase II and III AVF2107 trials, which confirmed the superiority of chemotherapy (IRI, 5-FU, and leucovorin) plus bevacizumab over chemotherapy plus placebo.^182^ Bevacizumab is a humanized IgG monoclonal antibody targeted to VEGF-A that, according to the AVF2107 trial, improves both PFS and OS in metastatic CRC (RR: 44% vs. 34.8%; OS: 20.3 vs. 15.6 months; HR: 0.66, *p* < 0.001; PFS: 10.6 vs. 6.2 months; HR: 0.54; *p* < 0.001). Therefore, the FDA-approved bevacizumab as the first VEGF-targeted agent for metastatic CRC, even though several trials investigating bevacizumab plus monotherapy or FOLFOX/FOXFIRI showed only a partial significant improvement in either OS or PFS.^182–187^ Using bevacizumab may lead to 10% more grade 3–5 adverse events, such as hypertension or leukopenia,^188^ while it remained relatively safe and effective when treating elderly patients with CRC (age over 70 years old) in the phase III AVEX trial.^185^ Further investigation found that both patients with KRAS mutations and those with a wild-type genotype may benefit from bevacizumab.^189–191^ Both left- and right-sided colon tumors respond well to bevacizumab.^191^ Two independent trials stated no difference in terms of efficacy against metastatic CRC between FOLFOX and FOLFIRI combined with bevacizumab.^192,193^ Yet interestingly, a bevacizumab-containing regimen seemed to have better efficacy with the triplet FOLFOXIRI regimen than FOLFIRI alone (PFS: 12.3 vs. 9.7 months; HR: 0.77; *p* = 0.006; OS: 29.8 vs. 25.8 months; HR: 0.80; *p* = 0.03), although the latter doublet regimen had fewer adverse reactions according to the TRIBE trial.^187^

In addition to first-line application of bevacizumab, various trials have validated its efficacy in the second-line setting. Longer PFS (7.3 vs. 4.7 months, HR = 0.61, *p* < 0.001) and OS (12.9 vs. 10.8 months, HR = 0.75, *p* = 0.0011), as well as a better response rate (22.7% vs. 8.6%, *p* = 0.0001), were seen in the E3200 trial with a combination of FOLFOX and bevacizumab than with FOLFOX alone for patients with CRC who progressed after FOLFOX therapy.^194^ Similar numerical differences were also noted in the comparison with bevacizumab alone. Even so, continuation on bevacizumab for those who progressed after first-line chemotherapy was still helpful for PFS (5.7 vs. 4.1 months, HR = 0.68, *p* < 0.001) and OS (11.2 vs. 9.9 months, HR = 0.81, *p* = 0.0062) improvement compared with standard chemotherapy alone in the phase III ML18147 trial.^195^

In terms of maintenance, that is, bevacizumab after first-line chemotherapy in stable CRC, a series of trials demonstrated that anti-VEGF agents might be quite attractive. The prospective and observational BRiTE study indicated that bevacizumab continuation dramatically improved the OS of patients with CRC (31.8 vs. 19.9 months, HR = 0.48, *p* < 0.001) in comparison with no maintenance.^196^ Continuation of CAP and bevacizumab significantly prolonged the progression time in patients after first-line XELOX plus bevacizumab compared with observation (11.7 vs. 8.5 months, HR = 0.67, *p* < 0.0001)^186^ regardless of RAS/BRAF mutation status and mismatch repair (MMR) status.^197^

Trends of longer OS (23.2 vs. 20.0 months, HR = 1.05, *p* = 0.65 in the MACRO trial and 25.4 vs. 23.8 months, HR = 0.83, *p* = 0.2 in the SAKK (Swiss Group for Clinical Cancer Research) trial) have been observed for maintenance bevacizumab plus XELOX over bevacizumab alone in the MACRO trial^198^ and for maintaining single-agent bevacizumab therapy compared with no treatment in the SAKK trial.^199^ No inferiority has been found for maintenance of bevacizumab alone over bevacizumab plus 5-FU or continuation of bevacizumab plus CAP over bevacizumab plus XELOX.^200,201^

#### Emerging anti-VEGFR agents

Until now, only bevacizumab has been FDA approved as a first- and second-line VEGF-targeted agent for CRC, although various novel agents are emerging, and some of them have been approved for second-line treatment of CRC.

Aflibercept is a VEGFR-1 and VEGFR-2 extracellular domain recombinant fusion protein that acts as a ligand trap targeting VEGF-A, VEGF-B, and PIGF. Aflibercept has a stronger affinity for VEGF-A than bevacizumab.^202^ The single-agent benefit of aflibercept seems to be limited,^202^ while chemo-combinations showed great potential according to the phase III VALOUR trial, in which the addition of aflibercept after OX or bevacizumab in metastatic CRC patients receiving FOXFIRI gained a better response (19.8% vs. 11.1%) as well as a longer PFS (6.9 vs. 4.7 months, HR = 0.76; *p* < 0.001) and OS (13.5 vs. 12.1 months, HR = 0.82; *p* = 0.0032) than FOXFIRI plus placebo.^203^ However, in terms of the first-line setting, as in the phase II AFFIRM trial, the combination of aflibercept with FOLFOX did not result in noticeable benefits in PFS or response rate, but did result in increased adverse event rates. Therefore, aflibercept should remain a second-line recommended CRC agent.^204^

Ramucirumab, a fully humanized monoclonal VEGFR-2-targeted IgG antibody, is another FDA-approved drug for second-line treatment of metastatic CRC based on the phase III RAISE trial. In this second-line-setting trial, a combination of ramucirumab and FOLFIRI significantly prolonged PFS (5.7 vs. 4.5 months; HR = 0.79, *p* < .0005) and OS (13.3 vs. 11.7 months, HR = 0.84, *p* = 0.022) compared with FOLFIRI-placebo.^205^ Similar to the findings with aflibercept, a phase II trial showed that the FOLFOX regimen may not benefit from addition to ramucirumab in terms of PFS.^206^

TKIs have become an appealing choice for patients with anti-EGFR-resistant NSCLC, while in patients with CRC, very few drugs have proven to be effective. Regorafenib, a TKI with multiple targets, such as VEGFR, PDGFR (platelet-derived growth factor receptor), FGFR (fibroblast growth factor receptor), and BRAF, was approved by the FDA to treat metastatic CRC. A first-line study concerning regorafenib plus FOLFOX in CRC found no improvement in the response rate compared with FOLFOX plus placebo.^207^ However, for refractory metastatic CRC treatment, in the phase III CORRECT trial,^208^ better median OS (6.4 vs. 5.0 months, HR = 0.77, *p* = 0.0052) and PFS (1.9 vs. 1.7 months, HR = 0.49, *p* < 0.0001) were achieved using regorafenib than using placebo, which has also been validated in an Asian population in the CONCUR trial (PFS: 3.2 vs. 1.7 months, HR = 0.31, *p* < 0.0001; OS: 8.8 vs. 6.3 months, HR = 0.55, *p* = 0.0002).^209^

Other agents are being developed quickly. The phase III FRESCO trial supported fruquintinib, a TKI with the ability to block VEGFR-1, VEGFR-2, and VEGFR-3, as a recommended choice for chemotherapy against refractory metastatic CRC. In this Chinese-based study, OS (9.3 vs. 6.6 months, HR = 0.65, *p* < 0.001) and PFS (3.7 vs. 1.8 months, HR = 0.26, *p* < 0.001) were significantly prolonged with fruquintinib compared with placebo,^210^ which led to approval of by the China Food and Drug Administration (CFDA) also known as NMPA (National Medical Products Administration). Famitinib is another TKI targeting the c-KIT receptor, VEGFR-2, and VEGFR-3, PDGFR, and RET that is being investigated in an ongoing phase II study, which has so far shown an improved PFS (2.8 vs. 1.5 months, HR = 0.58, *p* = 0.0034) and disease control rate (57.58% vs. 30.91%, *p* = 0.0023) for Famitinib, with results concerning OS waiting to be reported.^211^

New TKIs expressing remarkable antitumor effects in preclinical studies have produced unsatisfying OS and RR values in recent reports; however, PFS may be prolonged by drugs such as the VEGFR-2- and FGFR-targeted brivanib^212^ and cediranib, a TKI targeted to all three VEGFRs and PDGFR that failed to present efficacy towards CRC control in the phase II and III HORIZON study,^213,214^ as did nintedanib, a TKI with the ability to block all VEGFRs, FGFR1-3, PDGFR-α, and PDGFR-β, according to the phase III LUME-Colon 1 trial.^215^ Other on-market TKIs, such as the gastrointestinal stromal tumor (GIST)-targeted imatinib and sunitinib and the squamous cell carcinoma-targeted erlotinib and gefitinib, have no indication or supporting data for treating CRC. The major agents for antiangiogenic therapy under clinical investigation in CRC are summarized in Tables 3 and 4.

**Table 3:** Antiangiogenic agents in colorectal cancer

Agent	Key trial (NCT number)	Design (*N*)	Subject	Treatment	Main results		
					RR	OS	PFS
Bevacizumab	CAIRO-3^186,197^	Phase III (*N* = 558)	mCRC	Capecitabine + Beva	NA	25.9 m	11.7 m
	NCT00442637		First-line treatment		NA	(HR 0.83)	(HR 0.67***)
				Observation		22.4 m	8. 5 m
Bevacizumab	TRIBE^187^	Phase III (*N* = 508)	mCRC	FOLFOXIRI + Beva	NA	29.8 m	12.3 m
	NCT00719797						
				FOLFIRI + Beva		(HR 0.8*)	(HR 0.77**)
					NA	25.8 m	9.7 m
Bevacizumab	AVEX^185^	Phase III (*N* = 280)	mCRC	Beva + capecitabine x	19%	20.7 m	9.1 m
	NCT00484939					(HR 0.79)	(HR 0.53***)
				Capecitabine	10%	16.8 m	5.1 m
Bevacizumab	AVF2017^182^	Phase III (*N* = 813)	mCRC	Beva + IFL	44.8%	20.3 m	10.6 m
			Untreated			(HR 0.66**)	(HR 0.54**)
				Placebo + IFL	34.8%	15.6 m	6.2 m
Bevacizumab	ECOG3200^194^	Phase III (*N* = 829)	mCRC	FOLFOX + Beva	22.7%	12.9 m	7.3 m
			Second line			(HR 0.75**)	(HR 0.61***)
				FOLFOX	8.6%	10.8 m	4.7 m
				Beva	3.3%	10.2 m	2.7 m
Bevacizumab	ML18147^195^	Phase III (*N* = 810)	mCRC	Beva + Chemo^a^	2.8%	11.2 m	5.7 m
	NCT00700102		Second line	Chemo		(HR 0.81**)	(HR 0.68***)
					2.0%	9.8 m	4.1 m
Regorafenib	CONCUR^209^	Phase III (*N* = 204)	mCRC	Regorafenib	4%	8.8 m	3.2 m
	NCT01584830		Treatment refractory	Placebo		(HR 0.55***)	(HR 0.31***)
					0%	6.3 m	1.7 m
Regorafenib	CORRECT^208^	Phase III (*N* = 760)	mCRC	Regorafenib	1% (no CR)	6.4 m	1.9 m
	NCT01103323		Treatment refractory	Placebo		(HR 0.77**)	(HR 0.49***)
					0.4% (no CR)	5.0 m	1.7 m
Ziv-aflibercept	VELOUR^203^	Phase III (*N* = 1226)	mCRC	FOLFIRI + aflibercept	19.8%	13.5 m	6.9 m
	NCT00561470		Treatment refractory			(HR 0.817**)	(HR 0.76***)
				FOLFIRI + placebo	11.1%	12.06 m	4.67 m
Ramucirumab	RAISE^205^	Phase III (*N* = 1072)	mCRC	Ramucirumab + FOLFIRI	13.4%	13.3 m	5.7 m
	NCT01183780		Treatment refractory			(HR 0.844*)	(HR 0.793***)
				FOLFIRI + placebo			
					12.5%	11.7 m	4.5 m

*CRC* colorectal cancer, *mCRC* metastatic colorectal cancer, *RR* response rate, *OS* overall survival, *PFS* progression-free survival, *VEGF* vascular endothelial growth factor, *VEGFR* vascular endothelial growth factor receptor, *EGFR* epidermal growth factor receptor, *PDGFR* platelet-derived growth factor receptor, *FGFR* fibroblast growth factor receptor

**Table 4:** Antiangiogenic agents under clinical investigation

Name or ID	Targets	Condition	Phase	NCT identifier
LYN00101	VEGF	Solid tumors including CRC	Phase 1	NCT03644459
Vanucizumab	VEGF-A/angiopoietin-2	mCRC	Phase 2	NCT02141295
Sorafenib	VEGFR	mCRC	Phase 2	NCT03251612
		mCRC	Phase 2	NCT01471353
		Pretreated CRC	Phase 2	NCT01290926
		KRAS-mutated mCRC	Phase 2	NCT01715441
		mCRC	Phase 2	NCT00826540
		mCRC	Phase 2	NCT00865709
Linifanib	VEGFR	Advanced CRC	Phase 2	NCT00707889
Icrucumab	VEGFR	CRC	Phase 2	NCT01111604
Nintedanib	VEGFR	Refractory mCRC	Phase 3	NCT02149108
Vatalanib	VEGFR	mCRC	Phase 3	NCT00056446
		mCRC	Phase 3	NCT00056459
Semaxanib	VEGFR	mCRC	Phase 3	NCT00004252
		Advanced CRC	Phase 1/2	NCT00005818
Vandetanib	VEGFR/EGFR	CRC	Phase 2	NCT00454116
		mCRC	Phase 1	NCT00532090
		mCRC	Phase 2	NCT00500292
		Advanced CRC	Phase 1	NCT00496509
Famitinib	VEGFR-2/-3/PDGFR	Advanced CRC	Phase 2	NCT01762293
Tanibirumab	VEGFR-2	Advanced or metastatic cancer	Phase 1	NCT01660360
Cediranib	VEGFR-2	First-line mCRC	Phase 3	NCT00399035
		First-line mCRC	Phase 2/3	NCT00384176
		Solid tumors	Phase 2	NCT003851614
Brivanib	VEGFR-2/FGFR	KRAS-wild-type tumors and mCRC	Phase 3	NCT 00640471
LY3022856	VEGFR-3	Advanced solid tumors	Phase 1	NCT 01288989
Apatinib	VEGFR-2/c-Kit/ Src	Stage IIIB or IIIC CRC	Phase 3	NCT 03365765
		Second-line CRC	Phase 2	NCT 03271255
		Refractory CRC	Phase 2	NCT 03190616
Fruquintinib	Pan-VEGFR	Advanced CRC	Phase 4	NCT04005066
Tivozanib	Pan-VEGFR	First-line mCRC	Phase 2	NCT01478594
Motesanib AMG 706	Pan-VEGFR	mCRC	Phase 1	NCT 00101894
Sulfatinib	Pan-VEGFR	Advanced solid tumors	Phase 1	NCT 02133157
		Advanced solid tumors	Phase 1/2	NCT 02549937
Motesanib AMG 706	Pan-VEGFR	mCRC	Phase 1	NCT 00101894
Lenvatinib	Pan-VEGFR	Advanced solid tumors	Phase 2	NCT 03797326
Cabozantinib	Pan-VEGFR	Solid tumors	Phase 1/2	NCT03170960
		KRAS-wild-type mCRC	Phase 1	NCT02008383
Axitinib	Pan-VEGFR	First-line mCRC	Phase 2	NCT 01490866
		mCRC	Phase 2	NCT 00615056
Pazopanib	Pan-VEGFR	Second-line mCRC	Phase 1	NCT 00540943
		CRC	Phase 1	NCT00387387
Sunitinib	Pan-VEGFR	mCRC	Phase 3	NCT 00457691
MNRP1685A	Neuropilin-1	Solid tumors	Phase 1	NCT00747734
		Solid tumors	Phase 1	NCT00954642

*CRC* colorectal cancer, *mCRC* metastatic colorectal cancer, *VEGF* vascular endothelial growth factor, *VEGFR* vascular endothelial growth factor receptor, *EGFR* epidermal growth factor receptor, *PDGFR* platelet-derived growth factor receptor, *FGFR* fibroblast growth factor receptor

### Resistance to antiangiogenic therapy

Resistance to anti-VEGF has been observed in various cancer types, including CRC, which may be explained by compensatory activation of other signaling pathways and alternative excretion of angiogenesis-related proteins.

The fact that PIGF is upregulated and overexpressed in CRC cases that are resistant to antiangiogenic therapies^216^ suggests that PIGF is a crucial factor in overcoming anti-VEGF resistance, which might explain why aflibercept performed better than bevacizumab in xenograft models.^217^

The angiopoietin/TIE (tyrosine kinase with Ig-like and EGF-like domains) signaling RTK pathway contributes to vascular formation and stabilization by mediating downstream the RAS/RAF and PI3K/AKT pathways, which may be negatively regulated by angiopoietin-2. Abnormally increased levels of angiopoietin-2 have been noticed in a wide range of cancers, including CRC, and are associated with resistance to bevacizumab.^218^ Targeting both VEGF and angiopoietin-2 in preclinical studies helped control proliferation and progression in cancers that were resistant to VEGF-targeted therapies.^219–221^ The VEGF-A and angiopoietin-2 cotargeting agent vanucizumab, which inhibited growth in a CRC xenograft model,^222^ has passed through a phase I study with acceptable safety and encouraging anticancer effects.^223^

The FGF/FGFR pathway is important in both normal and cancer tissues for cell growth, survival, and migration. Upregulation of the FGF/FGFR pathway has also been observed in anti-VEGF-resistant cases.^224–226^ Dual blockade of FGF/FGFR and VEGF/VEGFR in preclinical studies displayed positive effects against tumor cells, while in clinical trials, agents such as nintedanib and the FGF-VEGF dual blocker dovitinib failed to benefit anti-VEGF-refractory patients.^215,227^

Compensatory activation of the c-MET pathway is the mechanism most related to the loss of anti-VEGF agent effectiveness.^228^ Single-agent c-MET inhibition might be helpful, as we shall discuss in the following section. However, CRC-based evidence for c-MET and VEGF dual targeting remains rare, and a study on NSCLC stated no better effect by combined blocking.^229^

A number of studies found factors such as a high level of TGF-β,^230,231^ upregulation of IL-1,^231^ downregulation of MIF (macrophage migration inhibitory factor),^232^ and overexpression of PDGFR^233^ in a wide range of VEGF-blockade-resistant cancers, implying possible connections to antiangiogenic therapeutic resistance; however, a lack of adequate data on silencing these factors in clinical cases has limited their further confirmation for CRC therapy.

### Anti-EGFR or antiangiogenic therapies?

Both anti-EGFR and antiangiogenic therapies have demonstrated decent effects against metastatic CRC; however, which one is the preferred first-line choice for a more precise and personalized targeted agent strategy has been a matter of intense debate. The first head-to-head comparison study was the phase III FIRE-3 trial, which compared bevacizumab and cetuximab in a combined regimen with FOLFIRI. No obvious difference was discovered in the response rate or PFS for both arms, yet OS was prolonged in the cetuximab arm (28.7 vs. 25 months, HR = 0.77, *p* = 0.017).^189^ Similar results were observed in a recent phase III trial investigating these two agents plus FOLFOX/FOLFIRI therapy, which reported few differences in the response rate, PFS, and OS between the two groups.^191^ The PEAK trial, focusing on panitumumab and bevacizumab with FOLFOX, stated that the response rate and PFS seemed alike, and a slightly longer OS for panitumumab than bevacizumab (34.2 vs. 24.3 months, HR = 0.77, *p* = 0.017) was noted.^190^ Further analysis in subgroups emphasized the importance of an individualized strategy. RAS mutation status might influence the efficacy of anti-EGFR therapy, but not that of anti-VEGF therapy; therefore, subgroup studies concerning gene information have been carried out. Cetuximab appeared to be the better choice for RAS-wild-type patients in the FIRE-3 post hoc analysis trial, given the increased rate of objective response (72.0% vs. 56.1%, *p* = 0.0029) and early tumor shrinkage (68.2% vs. 49.1%, *p* = 0.0005) that were achieved in the cetuximab arm in these patients.^234^ A recommendation that anti-EGFR over anti-VEGF is favored in RAS-wild-type patients has also been proposed via a meta-analysis that included the FIRE-3, CALGB, and PEAK trials.^235^ In addition, sidedness has been a critical factor that has marked impact on prognosis.^236^ Left-sided tumors responded more to cetuximab than to bevacizumab (38.3 vs. 28 months, HR = 0.63, *p* = 0.02), while those on the right side of the colon tended to behave oppositely (8.3 *vs.* 23 months, HR = 1.44, *p* = 0.28) in the FIRE-3 trial,^237^ which corresponded with subgroup findings from the CALGB study.^236^ Analysis of panitumumab supported the same side- and genetic-related trends.^238^ Even when BRAF-mutated cases are removed, right-sided cancer might still benefit little from anti-EGFR therapy.^238^ For second-line treatment, switching a bevacizumab maintenance strategy to cetuximab or panitumumab^239^ made no difference in patients with progressed RAS-wild-type CRC according to two independent phase II trials.

Given these data, patients with RAS-wild-type metastatic CRC with tumors on the left side of the colon are recommended to start first-line treatment with chemotherapy combined with anti-EGFR drugs, and anti-VEGF agents should be considered as an alternative choice at all times.

## The HGF/c-MET pathway

### About the pathway

Hepatocyte growth factor (HGF) and the receptor tyrosine kinase known as mesenchymal–epithelial transition factor (c-MET or MET) encoded by the MET proto-oncogene play vital roles in tumor proliferation, survival, metastasis, and acquired drug resistance.^240–244^ This signaling pathway was first discovered from TPR-MET fusion genes (translocated promoter region locus on chromosome 1 and MET sequence on chromosome 7) of a human osteosarcoma cell line in the 1980s, when HGF was also named scatter factor because it was initially isolated from rat platelets responsible for epithelial dispersal in organ healing and regeneration.^245–248^

HGF is secreted mostly from mesenchymal tissues and is currently the only known ligand for MET. Its tissue and serum expression levels are related to poor prognosis of patients with different malignant tumors, such as breast,^249^ esophageal,^250^ and gastric cancers,^251^ and especially CRC. Patients with advanced CRC have elevated serum HGF at diagnosis and decreased levels after cancer resection.^252,253^

MET is a member of the surface transmembrane receptor family expressed in both normal and malignant epithelial and endothelial cells, as well as in neural cells, hematopoietic cells, and hepatocytes.^254,255^ Overexpression of MET has been found in various carcinomas, such as hepatocellular carcinoma, lung cancer, breast cancer, thyroid cancer, kidney cancer, gastric cancer, and CRC.^256–262^ Increased mRNA and protein levels of MET were reported in CRC tissues, and its connection to tumor progression and metastasis was demonstrated in several studies.^263–265^

Activation of MET signaling starts from HGF binding to the MET receptor on the membrane, triggering the formation of an intracellular multifunctional docking site from two tyrosine residues, which bind to subsequent substrates. The activated HGF/MET pathway initiates various downstream signal transduction pathways, including the MAPK/ERK, PI3K/AKT, and STAT/JAK pathways and the nuclear factor-κB complex, to regulate hematopoiesis, organ regeneration, and wound healing.^244,252,254,255,266^ Gene amplification, overexpression, and mutation and ligand-dependent autocrine or paracrine signaling loops are commonly found in aberrant HGF/MET axes in oncogenic situations.^267^ Interestingly, MET mutations and amplifications are rarely discovered in patients with CRC, with rates of 2–5% and 0.5–2%, respectively.^119,268^ However, as mentioned above, overexpression of HGF/c-MET mRNA and protein was observed in over 70% and 50% of CRC tissue samples, respectively.^269–271^ The amplification of HGF-MET paracrine and autocrine loops was first identified by Boccaccio et al.^272^ Subsequent studies supported the theory that overactivated MET promotes enhanced HGF transcription and expression, thus contributing to subsequent MET expression to form a loop that can be further augmented through paracrine HGF produced by reactive stromal cells in the tumor microenvironment or under certain situations, such as hypoxia or inflammation.^273–277^

In addition to self-regulation, the HGF/MET signaling pathway might also be modulated by other factors. Plexin B family members have been reported to have structural similarity with MET. Activated plexin B1 might transactivate MET to modulate cancer growth and migration. However, the role of plexin B1 remains controversial because in various cancers, both tumor-promoting and tumor-suppressing effects have been observed.^278–280^ A recent study found that a newly identified gene, MACC1 (metastasis-associated in colon cancer 1), has high potential to be a key regulator of MET expression and further influence CRC progression and metastasis.^281^ Elevated levels of MACC1 expression were found in both local and metastatic malignant tissues. Accumulating data revealed HGF-induced MACC1 translocation from the plasma into nuclei, and MACC1 binding to the MET promoter contributed to enhanced MET transcription. MACC1 research provides new evidence for a positive loop of MET expression in CRC.

Another major method of regulating signaling activity relies on crosstalk between the MET pathway and other RTKs, especially EGFR. Overexpression of both MET and EGFR is commonly found in the same malignant tumor, such as CRC.^282^ Compensatory activity regains of MET or EGFR after targeted treatment of either of them has been observed in various studies, strongly implying the existence of crosstalk between MET and EGFR. As such, MET was the first factor to be identified as responsible for EGFR inhibitor resistance, even in the absence of known resistance-related mutations.^240,283–286^ Blocking either aberrant MET or aberrant EGFR leaves little restraint on downstream ERK or PI3K activation, while resistance abrogation was observed using combined therapy targeting both receptors in vivo and in vitro.^283,286,287^ Mutual regulation between MET and EGFR has several possible mechanisms. Downstream products of EGFR might induce phosphorylation of MET, whereas altered c-MET-induced protein may also lead to EGFR phosphorylation.^282,287^ Activated MET and EGFR might form different heterodimers, resulting in various tumor biological behaviors, such as cell growth and survival for MET-EGFR and MET-HER3 or migration for MET-HER2.^288^ In addition, MET activation has been observed occasionally in VEGF-targeted therapy, resulting in VEGF resistance; however, the underlying mechanism remains unclear.^289,290^

### Targeting the HGF/c-MET pathway

Accumulating knowledge of the close relationship between cancer and the HGF-MET pathway identifies it as a highly promising site for targeted therapy. Various ways of blocking HGF-MET via newly developed monoclonal antibodies or small molecules with different pharmacological mechanisms have emerged. For HGF, drugs are aimed at either blocking HGF activation and production or interfering with the binding of HGF to MET receptors. In the latter case, agents either competitively bind to MET receptors (MET antagonists) or inhibit intracellular tyrosine kinase activity (MET TKIs). To date, no severe adverse events have been reported for these agents, although some patients complained about fatigue, poor appetite, allergic reactions, edema, skin rash, and neutropenia.^252,266^ There are several current clinical trials of HGF/c-MET-targeted agents in the context of CRC therapy (Table 5).

**Table 5:** HGF-MET-targeted agents under clinical investigation

Name or ID	Targets	Condition	Phase	NCT identifier
Rilotumumab	HGF	Gastrointestinal cancer	Phase 1/2	NCT00788957
TAK-701	HGF	Advanced solid tumors	Phase 1	NCT00831896
Onartuzumab	MET	CRC	Phase 2	NCT01418222
ABT-700	MET	Advanced solid tumors	Phase 1	NCT01472016
ABBV-399	MET	Solid tumors	Phase 1	NCT02055066
YYB-101	MET	Solid tumors	Phase 1	NCT02499224
ARGX-111	MET	Solid tumors	Phase 1	NCT02099058
Tivantinib	MET	mCRC	Phase 1/2	NCT01075048
Savolitinib	MET	mCRC	Phase 2	NCT03592641
AMG 337	MET	Advanced solid tumors	Phase 1	NCT01253707
Capmatinib	MET	Multiple tumors including CRC	Phase 1	NCT02386826
Crizotinib	MET/RON/ROS	Solid tumor	Phase 1	NCT02510001
		Solid neoplasm	Phase 2	NCT02465060
Cabozantinib	MET/RET/VEGFR-2	CRC	Phase 1	NCT02008383
		CRC	Phase 1	NCT03539822
		CRC	Phase 1	NCT03798626
		Multiple tumors including CRC	Phase 1/2	NCT03170960
		CRC	Phase 2	NCT03542877
Foretinib	MET/VEGFR	Solid tumors	Phase 1	NCT00742261
		Solid tumors	Phase 1	NCT00743067
Golvatinib	MET/VEGFR	Solid tumors	Phase 1/2	NCT01355302
Merestinib	MET/TEK/ROS1/DDR/MKNK	Multiple tumors including CRC	Phase 1	NCT02745769
Sitravatinib	MET/VEGFR/DDR	Advanced tumors	Phase 1	NCT02219711
		Advanced tumors	Phase 1	NCT03666143

*CRC* colorectal cancer, *mCRC* metastatic colorectal cancer, *HGF* hepatocyte growth factor, *MET* mesenchymal–epithelial transition factor, *TEK* tunica interna endothelial cell kinase, *DDR* discoidin domain receptor tyrosine kinase, *MKNK* MAP kinase-interacting serine/threonine protein kinase, *FLT3* Fms-related tyrosine kinase 3, *VEGFR* vascular endothelial growth factor receptor, *MEK* mitogen-activated protein kinase

#### HGF inhibitors

HGF production relies on the maturation of its precursor (pro-HGF), mediated by an endogenous inhibitor protein family called HGF activator inhibitors (HAIs).^291,292^ High levels of HAI-1 have been observed in patients with benign lesions compared with those with prostate cancer, and the level of HAI-2 was decreased in highly invasive and progressed prostate cancer cells.^293,294^ The HAI protein family seems to be an attractive target to control HGF activation; however, no artificial compound or analog has been made so far, and thus, clinical testing is a long way off, although experimental data imply a potential role in an antimetastatic strategy for CRC.^295,296^

Instead of blocking HGF activation, neutralizing HGF to impede its ability to bind to receptors to interfere with whole pathway appears to be more practical. A few monoclonal antibodies have been synthesized and introduced in several clinical trials. Rilotumumab, a humanized IgG monoclonal antibody, has been investigated in phase I and II trials. In those patients with gastric or gastroesophageal cancer, a prolonged median PFS (6.8 vs. 4.4 months; HR = 0.46, 95% confidence interval (CI): 0.25–0.85) and OS (10.6 vs. 5.7 months; HR = 0.46, 95% CI: 0.24–0.87) were achieved in patients with MET overexpression using rilotumumab plus CAP compared with those in the placebo plus CAP arm.^297^ Further phase III studies (RILOMET-1 and RILOMET-2)^261,298^ in patients with untreated or advanced-stage gastric or gastroesophageal cancer were halted early because of a rapid increase in disease-related deaths. These trials highlighted the importance of stratification. Current trials commonly apply methods such as IHC or FISH to determine the existence of MET overexpression, and a further scoring system according to the percentage of tumor cells with high staining intensity is used to stratify MET-positive/high and MET-negative/low patients, although the criteria differ by small degrees.

For patients with CRC, a randomized phase Ib/II trial^299^ concerning rilotumumab or ganitumab vs. panitumumab in patients with KRAS-wild-type metastatic CRC showed no significant benefit with the combined use of rilotumumab and panitumumab in terms of median OS (13.8 vs. 13.7 months, *p* = 0.71) in patients with MET-high disease compared with MET-low disease.

Ficlatuzumab is a humanized IgG monoclonal antibody that has been investigated in a phase I trial for advanced solid tumors and liver metastases.^300^ TAK-701, another humanized anti-HGF monoclonal antibody, was combined with gefitinib to help overcome EGFR resistance in lung cancer and is also undergoing a phase I trial.^301–303^

#### MET antagonists

Agents that compete with HGF for binding to MET result in abnormal dimerization and degradation of MET. Various antibodies have been developed, including onartuzumab, DN-30, and ABT-700. Onartuzumab, a murine-derived monoclonal antibody with high specificity for the MET semaphorin domain,^304^ has been evaluated in several trials in patients with solid tumors, such as NSCLC, glioblastoma, gastroesophageal cancer, gastric cancer, and CRC.^305–308^ Improved median OS and PFS were observed in MET-positive lung cancer patients treated with erlotinib in a phase II trial; however, no such efficacy was reported in a phase III trial.^306^ Similarly, in gastric or gastroesophageal cancer, no significant improvement in PFS or OS was observed using onartuzumab plus mFOLFOX6 vs. placebo plus mFOLFOX6.^305^ In the case of metastatic CRC, no significant differences were identified in PFS between MET-positive and MET-negative patients using onartuzumab combined with mFOLFOX6 + bevacizumab or placebo.^308^

There are a few novel MET antibodies that function promisingly in cancer control yet lack supporting data in CRC. The antibody DN-30 binds to the IPT (Ig-like, plexins, transcription factors) domain of MET and shows a promising ability to inhibit the proliferation of MET-positive gastric cancer and metastatic melanoma in vitro and in vivo.^309^ ABT-700 is a humanized antibody that could induce gastric and liver tumor regression in preclinical cancer models with MET amplification and passed a phase I trial in several solid tumors with favorable safety and tolerability.^310–312^ Emibetuzumab, a humanized antibody targeting MET, has been used in phase I and II trials for NSCLC and gastric cancer.^313–316^ Other anti-MET agents, such as ABBV-399, YYB-101, and ARGX-111, are either undergoing or just past phase I trials for a range of solid tumors.

#### MET TKIs

A number of drugs functioning as selective or nonselective TKIs have been developed and brought to clinical trials. To some extent, their similar RTK structure to MET guarantees their pharmacological effects. Selective agents include tivantinib (ARQ 197), savolitinib (AZD 6094; volitinib), AMG 337, and capmatinib (INC 280), which target the MET kinase domain, and nonselective agents include crizotinib (PF-02341066), cabozantinib (XL-184), tepotinib (EMD-1214063), foretinib (GSK1363089), glesatinib (MGCD-265), golvatinib (E-7050), and merestinib (LY-2801653).

Tivantinib is an oral small-molecule allosteric RTK inhibitor that selectively keeps MET in the inactive state. In vivo and in vitro experiments confirmed its ability to impair the growth of various cancers.^317–319^ Tivantinib has been investigated as an independent drug or combined with sorafenib in phase II and III trials in patients with liver cancer and combined with erlotinib in phase III trials in patients with NSCLC. Data showed that patients with liver cancer with MET overexpression had a better OS than those without MET overexpression in a phase II trial, but the significance was lost in a phase III trial, while the use of tivantinib contributed little to the prognosis of NSCLC.^320,321^ For patients with metastatic CRC, a phase I/II trial enrolling patients to receive tivantinib or placebo plus cetuximab and IRI found no PFS improvement,^322,323^ and a phase II trial concerning tivantinib or placebo plus cetuximab did not meet its primary end point because of a low response rate.^324^ To date, various MET-specific TKIs have shown dramatic effects in treating several malignancies, such as NSCLC or gastric cancer, and have been used in phase I clinical trials. However, clinical trials on CRC are insufficient to evaluate the efficacy of tivantinib and other selective TKIs.

AMG 337, an oral ATP-competitive TKI specific to MET, caused a strong response in patients with MET-amplified upper gastrointestinal tract cancer in phase I and II trials.^325,326^ Savolitinib, a selective MET inhibitor, displayed marked antitumour potential under experimental conditions and appeared to be effective against renal cell cancer^327,328^ and is being investigated in a metastatic CRC phase I trial. Capmatinib, another selective MET inhibitor, has been demonstrated as a good supplementary agent to gefitinib in patients with EGFR-mutant, MET-amplified NSCLC.^329^

Nonselective TKIs targeting multiple factors, including the TKs of MET, may have a wider range of applications.

Crizotinib targets anaplastic lymphoma kinase (ALK) and the TKs of MET, and the RON (macrophage-stimulating 1 receptor) and ROS (ROS proto-oncogene 1, receptor tyrosine kinase) receptors and is an FDA-approved treatment for ALK-rearranged NSCLC.^330^ Crizotinib has attractive efficacy in prolonging the survival of patients with NSCLC.^331^ Although there is a lack of clinical evidence for crizotinib in CRC, a series of phase I and II trials are in progress. The use of crizotinib might enhance the response to radiation therapy in KRAS-mutant CRC cell lines, and a combination of crizotinib with mitomycin C seemed to have a synergistic effect against CRC in preclinical results, which showed promise for future anti-CRC treatments.^332^

Cabozantinib, specific for a wide range of TKs, such as MET, RET, and VEGFR-2, is also an FDA-approved drug for metastatic medullary thyroid cancer and renal carcinoma, with antitumor effects in liver cancer, as assessed through phase II and phase III trials.^333–335^ Similarly, an exciting antitumor performance of cabozantinib was observed in research based on CRC xenograft models and cell lines, while the results from CRC-focused clinical trials are awaited.^289,336,337^

Merestinib, an inhibitor of MET, AXL (Axl receptor tyrosine kinase), TEK (tunica interna endothelial cell kinase), ROS1, DDR (discoidin domain receptor tyrosine kinase), MKNK (MAP kinase-interacting serine/threonine protein kinase), and FLT3 (Fms-related tyrosine kinase 3), has just finished its first human phase I trial for various advanced cancers, including CRC,^338^ to determine a suitable dose for the phase II trial.

TKIs are being upgraded rapidly from generation to generation. Although highlighted in preclinical studies or phase I/II trials for solid tumors such as gastric cancer or NSCLC,^339–343^ new agents such as tepotinib, foretinib, glesatinib, golvatinib, and sitravatinib are only a few steps from clinical investigation for CRC.

As described above, owing to the crosstalk between HGF/MET and other major pathways targeted by most targeted agents, targeting HGF/MET could help overcome resistance against EGFR or VEGFR inhibitors. Combined inhibition of MET and EGFR showed improved PFS in patients with NSCLC with MET overexpression; however, the study was halted prematurely because of an increased rate of interstitial lung disease.^344^ Similar antitumor effects have been noted in renal cancer xenografts treated with therapies targeted at both VEGF and MET.^345^

Although clinical evidence of HGF/MET-targeted drug resistance has not been presented, a preclinical study observed acquired resistance to HGF/MET inhibitors in patients with gastric cancer.^346^ Under laboratory conditions, suppression of HGF/MET might induce subsequent MET mutations (Y1230 mutation in the MET loop) and compensatory activation of alternative pathways, such as EGFR or the RAS/RAF/MEK pathway, which might inevitably cause secondary resistance to anti-HGF or anti-MET therapies.^277^

## Immune checkpoint inhibitor therapy

### About the pathway

In addition to methods directly blocking pathways that contribute to tumor growth and spread, accumulating data suggest that targeting other pathways to enhance immunorecognition and the response against cancer cells might be effective. Malignancies harboring various genetic and epigenetic alterations may be identified and obliviated by the host immune system via the expression of abnormal antigens. The detection process comprises several steps, including T cells binding to major histocompatibility complex (MHC) molecules held by antigen-presenting cells (APCs), followed by the next step involving secondary signals mediated via costimulatory or inhibitory receptors that play a vital role in the activation and tolerance of T cells.^347,348^ This double-check mechanism is important both in physiological conditions to avoid an excessive immune response and in pathological conditions so that abnormal cells may be attacked in a flexible manner.^349^

Immune escape, which refers to cancer cells escaping from host immunorecognition and response, has been frequently identified in various cancers.^350^ Secretion of immunosuppressive factors, such as TGF-β and IL-6, recruitment of immunosuppressive cells, such as regulatory cells, or loss of immunogenicity via downregulation of MHC-1 might all contribute to immune escape.^351–355^ One more major explanation is tumor-related T cell inactivation and exhaustion via activation of coinhibitory receptors, the so-called immune checkpoint receptors, on the surface of T cells,^356^ which include programmed death-1 (PD-1), or CD279, and cytotoxic T lymphocyte antigen 4 (CTLA-4). PD-1, with its ligands PD-L1 (CD274) and PD-L2 (CD 273), is a peripheral immune checkpoint on tumor, stromal, and immune cells, while CTLA-4 is a central checkpoint targeting B7-1/B7-2 or CD80/CD86 on APCs.^348,351,357^ Engaged CTLA-4 downregulates IL-2 secretion and competitively binds to B7-1/B7-2 to diminish the CD28-derived stimulatory effect of T cells, and activated PD-1 leads to inhibition of downstream pathways, such as the PI3K/AKT pathway, resulting in both the abrogation of T cell proliferation and eventual immune anergy.^358,359^ Like other mutation-enriched cancers, metastatic CRC lesions express higher levels of PD-L1 than primary lesions,^360^ laying the foundation for interfering with the host immune response.

Immune cells, including B cells and T cells, infiltrating into the CRC microenvironment were found to be highly related to alterations in the adaptive immune response and antitumor outcomes.^361,362^ Among them, CD4+ T regulatory (Treg) cells, which suppress the activation and function of other immune cells, were found to infiltrate into CRC tissues and related lymph nodes in vitro and in vivo.^363,364^ Indeed, in paired patient samples from CRC tissues and normal mucosa, increased numbers of Treg cells were observed in the CRC tissues.^365^ These PD-1-expressing Treg cells are considered crucial in the immune tolerance and homeostasis of CRC. Moreover, MHC-1-loss-detecting NK cells, which are effective in killing tumors that use downregulation of MHC-1 as a method of immune escape, were found to be dysfunctional in CRC, also partially because of the PD-1/PD-L1 interaction.^366,367^ Preclinical studies found T cell activation and inactivation of tumor cells when blocking the PD-1/PD-L1 interaction or CTLA-4.^368–370^ In addition, checkpoint inhibitors displayed promising effects against different advanced cancers, such as melanoma, renal cell cancer, and NSCLC, in recent phase III trials.^371–376^ These data provide the basis for the development of immune checkpoint-targeting therapy against CRC.

### Immune checkpoint blockade agents

Immune checkpoint targeted therapy aims to enhance immune surveillance and suppression against cancer by blocking the tumor’s attempt to escape from T cell detection.^377,378^ Currently, checkpoint inhibitors have been investigated in various solid tumors with promising responses. The first FDA-approved therapy was a CTLA-4 inhibitor, ipilimumab, for melanoma,^379–381^ followed by the PD-1 inhibitor nivolumab, which displayed significant effects with tolerable adverse events in a phase I trial and went through subsequent phase II and III trials. Another PD-1 inhibitor, pembrolizumab, was trialed for several advanced tumors, such as renal cell cancer, melanoma, and NSCLC, which led to its approval by the FDA for treating these malignancies.^371–376,382^

In metastatic CRC, although a significant response was noticed in some phase I trials,^383^ further studies found that only a small proportion of patients with CRC responded to immune checkpoint therapy.^384^ This subgroup of patients had a high tumor mutational burden and their malignant lesions showed high levels of microsatellite instability (MSI-H) or MMR deficiency (dMMR).^385–387^ Tumor mutational burden has been observed to be associated with the immune checkpoint response rate in melanoma and NSCLC;^388,389^ however, the underlying mechanism remains unclear. One hypothesis argued that tumor-related neoantigens derived from mutations favored attention from immune cells and thus attracted increased T cell infiltration.^390–392^

#### Pembrolizumab and nivolumab

The first PD-1 blocker to display good efficacy against MMR-deficient CRC was pembrolizumab, a humanized IgG4 antibody that started a new chapter in CRC immunotherapy when the FDA approved it for metastatic CRC treatment in 2017. The KEYNOTE-016 study found that patients with dMMR CRC might respond to pembrolizumab treatment (response rate of 40% and a 20-week PFS of 78%), while none of those with pMMR responded to the drug (response rate of 0% and a 20-week PFS of 11%).^387^ Another phase I trial also identified the antitumor activity of pembrolizumab only in patients with MSI-H CRC.^393^ Thus, the KEYNOTE-164 study investigated pembrolizumab in patients with MSI-H metastatic CRC in the second-line setting, which showed an objective response rate of 33% and PFS and OS values of 2.3 and 31.4 months, respectively.^394,395^ Further updated analysis of the data from KEYNOTE-016 demonstrated an objective response rate of 52%, a 2-year PFS of 59%, and an OS of 72%.^396^ Combined therapy with pembrolizumab and ipilimumab showed comparable efficacy in melanoma patients, but there is insufficient evidence in CRC.^397^

Nivolumab, another humanized monoclonal IgG4-based PD-1 antibody, gained FDA approval for dMMR or MSI-H metastatic CRC in 2017 based on the CheckMate-142 trial, in which 51 out of 74 patients enrolled achieved disease control for at least 12 weeks, with an objective overall response rate of 31.1%, regardless of the tumor PD-L1 level, and 1-year PFS and OS values of 50.4% and 73.4%, respectively.^382^ Further studies argued that a combination of nivolumab and the CTLA-4 inhibitor ipilimumab surpassed single-agent immune checkpoint blocking with an acceptable rate of adverse events.^398–400^ Combined therapy with nivolumab and ipilimumab helped patients with dMMR or MSI-H CRC who had previously received chemotherapy to reach a PFS of 71% and an OS of 85% at 1 year; 80% of them maintained disease control over 12 weeks. For first-line use of this doublet regimen, a new study assessed its efficacy and safety for patients with dMMR or MSI-H CRC.^399^ According to this trial of 45 patients, the 1-year PFS and OS values for the combined regimen were 77% and 83%, respectively, with an ORR of 60% and a disease control rate of 84%. This evidence gained the nivolumab and ipilimumab-containing doublet regimen FDA approval as a therapy for patients with chemotherapy-refractory metastatic CRC (Table 6).

**Table 6:** Main agents for immune checkpoint blockade therapy in colorectal cancer

Agent	Key trial (NCT number)	Design (*N*)	Subject	Main results		
				RR	OS (12 m)	PFS (12 m)
PD-1 plus CTLA-4 inhibitor						
Nivolumab + ipilimumab	CheckMate-142^382,398^	Phase II (*N* = 119)	dMMR/MSI-H mCRC	55%	85%	71%
	NCT02060188		Treatment refractory			
	CheckMate-142^382,399^	Phase II (*N* = 45)	dMMR/MSI-H mCRC	60%	83%	77%
	NCT02060188		Treatment refractory			
PD-1 inhibitor						
Nivolumab	CheckMate-142^382^	Phase II (*N* =74)	dMMR/MSI-H mCRC	31.1%	73.4%	50.4%
	NCT02060188		Treatment refractory			
Pembrolizumab	KEYNOTE-164^394,395^	Phase II (*N* = 61)	dMMR/MSI-H mCRC	33%	31.4 m	2.1 m
	NCT02460198		Treatment refractory			

*mCRC* metastatic colorectal cancer, *RR* response rate, *OS* overall survival, *PFS* progression-free survival, *dMMR* deficient mismatch repair, *MSI-H* microsatellite instability-high, *CTLA-4* cytotoxic T lymphocyte-associated antigen 4, *PD-1* programmed death-1, *m* months

#### Other agents

Other novel PD-1/PD-L1 inhibitors are under investigation (Table 7), including atezolizumab, avelumab, and durvalumab, which are IgG monoclonal antibodies targeting PD-L1 (BP 545758), some of which have been approved for the treatment of NSCLC and urothelial cancer. These agents have been through phase I trials for various solid tumors, including CRC, with manageable safety profiles and are undergoing further exploration. New immune checkpoint targets are being investigated under experimental conditions and are being evaluated for their safety profiles in phase I trials, such as tumor-overexpressed TIM-3 (T cell Ig and mucin domain-containing protein 3), T cell Ig, and ITIM domains (TIGIT), and T cell-derived LAG-3 (lymphocyte activation gene 3), which both contribute to T cell exhaustion and promote CRC progression.^401,402^

**Table 7:** Immune checkpoint modulators under clinical investigation

Name or ID	Targets	Condition	Phase	NCT identifier
Atezolizumab	PD-1/PD-L1	mCRC	Phase 3	NCT02788279
		Stage 3 CRC	Phase 3	NCT02912559
		First-line mCRC	Phase 3	NCT02997228
		Refractory CRC	Phase 2	NCT02873195
		First-line mCRC	Phase 2	NCT02291289
		mCRC	Phase 1	NCT02876224
Avelumab	PD-1/PD-L1	mCRC	Phase 2	NCT03150706
		mCRC	Phase 2	NCT03258398
PDR-001	PD-1/PD-L1	First-line mCRC	Phase 1	NCT03176264
		mCRC	Phase 1	NCT03081494
SHR-1210	PD-1/PD-L1	CRC/HCC/NSCLC	Phase 1	NCT03601598
JS-001 Toripalimab	PD-1/PD-L1	CRC	Phase 1/2	NCT03946917
		CRC	Phase 2	NCT04118933
		mCRC	Phase 2	NCT03927898
AMP-224	PD-1/PD-L1	CRC	Phase 1	NCT02298946
TSR-033	PD-1/PD-L1	CRC	Phase 1	NCT03250832
Camrelizumab	PD-1/PD-L1	mCRC	Phase 2	NCT03912857
AB-122	PD-1/PD-L1	Multiple tumors including CRC	Phase 1	NCT03629756
		Multiple tumors including CRC	Phase 1	NCT03628677
INT230-6	PD-1/CTLA-4	Multiple tumors including CRC	Phase 1/2	NCT03058289
ONC-392	CTLA-4	Multiple tumors including CRC	Phase 1/2	NCT04140526
Tremelimumab	CTLA-4	mCRC	Phase 1/2	NCT03202758
		CRC	Phase 1/2	NCT03206073
		mCRC	Phase 2	NCT03122509
		mCRC	Phase 2	NCT03428126
		mCRC	Phase 2	NCT02811497
		mCRC	Phase 2	NCT03122509
		mCRC	Phase 2	NCT03435107
		mCRC	Phase 1/2	NCT03202758
MGD-013	PD-1/LAG-3	Solid tumors	Phase 1	NCT03219268
Relatlimab	LAG-3	Solid tumors	Phase 1/2	NCT01968109
		CRC	Phase 2	NCT03642067
TSR-033	LAG-3	Solid tumors	Phase 1	NCT03250832
IMP-321	LAG-3	Solid tumors	Phase 1	NCT03252936
		Solid tumors	Phase 1	NCT02676869
REGN-3767	LAG-3	Solid tumors	Phase 1	NCT03005782
TSR-022	TIM-3	Solid tumors	Phase 1	NCT02817633
MBG-453	TIM-3	Advanced malignancies	Phase 1	NCT02608268
AB-154	TIGIT	Multiple tumors including CRC	Phase 1	NCT03628677
KRN-330	A33 glycoprotein	A33-positive colorectal cancer	Phase 1	NCT00575562
I-huA33	A33 glycoprotein	A33-positive colorectal cancer	Phase 1	NCT00291486

*CRC* colorectal cancer, *mCRC* metastatic colorectal cancer, *CTLA-4* cytotoxic T lymphocyte-associated antigen 4, *PD-1/PD-L1* programmed death-1/programmed death ligand 1, *TIM-3* T cell immunoglobulin and mucin domain-containing protein 3, *TIGIT* T cell immunoglobulin and ITIM domains, *LAG-3* lymphocyte activation gene 3

### Overcoming resistance to immunotherapy

Given the unsatisfactory results for immune checkpoint blockade therapy observed in patients with MMR proficient (pMMR) or microsatellite stable (MSS) CRC, who constitute the major proportion of patients with CRC, it is unfortunate that the underlying mechanism has not been clearly determined. Investigators have tried to overcome the resistance of pMMR or MSS CRC to immune checkpoint inhibitors on the basis of several hypotheses related to reduced tumor-specific antigen expression, antigen presentation defects, altered immunosuppressive pathways (e.g., activation of MAPK and loss of PTEN), and alternative activation of other immune checkpoint signaling pathways, immune regulatory cells, and cytokines.^403^ Strategies to improve pMMR or MSS CRC immune checkpoint inhibitor responses are being developed, such as combined therapy with various approaches including radiotherapy, bispecific antibody therapy, other immune checkpoint modulators, and other targeted agents.^377^

Radiotherapy might lead to upregulated expression of tumor-specific neoantigens through cell damage and increase membrane MHC class I expression, thus activating the host immune response.^404^ Radiotherapy combinations showed antitumor effects with well-tolerated adverse events in melanoma treatment, and this strategy seemed to benefit only the combined use of PD-1 and CTLA-4 blockers because the effects of a single agent might be overcome by upregulation of the other signaling pathway.^405^ For MSS CRC, several studies are ongoing that introduce radiotherapy into a doublet regimen with immune checkpoint-blocking therapy (NCT03104439, NCT03007407, and NCT02888743).

Another method to enhance T cell surveillance is the use of a bispecific antibody such as the CEA-TCB antibodies RG7802 or RO6958688, which bind to CEA on tumor cells and CD3 on T cells, thereby helping T cells infiltrate and identify tumor cells. Preclinical studies and a phase I trial showed that the CEA-TCB antibody plus atezolizumab had anti-MSS CRC potential with acceptable toxicity.^406–409^ Among various combinations of immune checkpoint inhibitors and other targeted agents, MEK blockers seem to have attracted increased attention because MEK blockade is linked to an increased T cell response via upregulation of PD-L1 expression.^410^ Trials have been conducted for combined blocking of MEK and immune checkpoints inspired by the phase I and III studies focused on atezolizumab and cobimetinib (a MEK inhibitor), which found that this regimen was well tolerated yet offered no significant survival improvement over single drugs such as regorafenib or TAS-102 in patients with MSS CRC.^411,412^ Strategies that interfere with other pathways, such as VEGF/VEGFR blockade with PD-1/PD-L1 inhibition, are being investigated in a wide range of trials, and a phase I study has verified the safety of the combined method.^413^

### Biomarkers for treatment surveillance

Considering that efficacy varies for immune checkpoint therapy and overdosing might lead to unwanted adverse events, identifying biomarkers for potentially sensitive patients and predicting their response becomes a vital task.

The PD-L1 expression level appeared to be a persuasive marker because PD-L1-positive lesions were more vulnerable to PD-1 inhibition therapy than PD-L1-negative lesions, yet clinical survival data did not show a significant relationship.^414–417^ The predictive role of PD-L1 expression in CRC is considered to be limited because in pMMR CRC, no obvious trend was observed between PD-L1 expression levels and drug efficacy.^382,387^

A high mutational burden correlates with elevated levels of neoantigens. It is not just dMMR and MSI-H tumors that harbor high mutational burdens. MSS and pMMR lesions might present with an ultramutated phenotype, such as the DNA polymerase epsilon (POLE) mutations that are found in ~1–2% of pMMR CRCs, which cause increased immunogenicity and upregulation of immune checkpoint genes such as PD-1/PD-L1 and CTLA-4, resulting in similar clinical responses to those seen in dMMR tumors.^418–420^ Only a few cases of POLE mutations indicating the efficacy of PD-1 blockade therapy have been reported; therefore, immune checkpoint modulator-based phase II or II trials enrolling larger groups of patients with POLE-mutated CRC have been initiated (NCT03150706, NCT03435107, and NCT03827044). Currently, whether genetic alterations contribute to high mutational burdens, other than MMR- and POLE-related alterations, remains uncertain, and whether the cost of testing the mutational load via methods like next-generation sequencing is justified might also be a matter of debate.^421^

In addition to immunorecognition, T cell infiltration is also fundamental for PD-1-blocking therapy; thus, the immunoscore based on the calculation of two lymphocyte populations (CD3/CD45-CD8 or CD8/CD45 populations) in the center and invasive margins of the tumor has been attractive for predicting the drug response and has acted as a reliable CRC classifier and recurrence estimator; however, it is waiting for further clinical validation.^422–424^

Some other factors indicating cytotoxic T cell activity, such as the level of granzymes, perforins, and IFN-γ, remain in the theoretical phase,^403^ and genetic PD-1 resistance-predicting markers such as β-2-microglobulin (B2M), JAK1, and JAK2, which have been validated in other tumors, such as melanoma, have not been confirmed to have similar effects in CRC, although the absence of B2M might correlate with a better clinical outcome.^425,426^

## Adjuvant and neoadjuvant therapy with targeted agents

### Targeted agents in adjuvant therapy

Fluoropyrimidine-based chemotherapy after curative surgery for CRC might help to minimize tumor recurrence and prolong survival. Adjuvant chemotherapy benefited patients with stage II cancer to a lesser degree than it benefited those who had tumors in stage III, suggesting that it might achieve better disease-free survival (DFS) and OS, as the former group typically had better prognosis, with a 5-year survival rate of almost 80% according to the randomized QUASAR study and the MOSAIC and NSABP C07 studies.^427–430^ MSI-H stage II CRC appeared to not respond to adjuvant chemotherapy.^431^ High-risk stage II tumors, such as those at stage T4, those with lymphovascular or perineural invasion, and those with perforation or obstruction; MSS tumors; and tumors in stage III were recommended to be treated with adjuvant FOLFOX or CAPOX therapy, which may reduce the 10–22% death rate.^102,432^ Notably, extra drug-derived toxicity should be taken into consideration^433^ in adjuvant therapy; thus, additive agents such as targeted drugs have been used to attempt to improve adjuvant treatment. Therefore, several subsequent trials have been initiated to refine current adjuvant therapy with previously proven targeted agents.

The phase III NSABP-C08 trial,^434^ AVANT trial,^435^ and QUASAR 2 trial^436^ aimed to introduce bevacizumab into either CAP, XELOX, or FOLFOX adjuvant regimens; however, none of them showed significantly prolonged DFS for patients with CRC, and only an increased rate of treatment-related adverse events was reported. A similar conclusion was drawn that no survival benefit could be gained from adjuvant application of cetuximab with the classic regimens FOLFOX or FOLFIRI, and for subgroup analysis of RAS- and BRAF-wild-type patients, an adjusted HR of 0.76 (*p* = 0.07) was noted, implying that a further larger randomized controlled study may be required.^437–439^ Moreover, the cell surface glycoprotein 17-1A antibody edrecolomab, which was once of high potential interest for use against CRC, also failed to improve adjuvant therapy.^440,441^ Based on these data, targeted agents have not been included in current adjuvant therapy; however, trials concerning immune checkpoint inhibitors are ongoing. Specifically, atezolizumab plus adjuvant FOLFOX for patients with dMMR or MSI-H CRC (ATOMIC trial: NCT02912559) and avelumab plus an adjuvant 5-FU-based regimen in patients with MSI-H or POLE-mutant CRC (POLEM trial: NCT03827044) are being studied.^442,443^

### Targeted agents in neoadjuvant therapy

Typically, neoadjuvant therapy should be applied for colon cancer with potential resectable metastases and rectal cancer of stages II to IV to minimize local recurrence. Routine approaches include radiation, chemotherapy, or combined chemoradiation. Reaching a pathological complete response (pCR), which indicates complete tumor remission, has always been the main goal of neoadjuvant therapy for rectal cancer, enabling possible sphincter-saving curative surgery with a reduced recurrence rate of 5–6%;^444–448^ however, current statistics showed an unsatisfying pCR rate of less than 15%.^448^

Targeted agents are considered to be novel weapons to enhance neoadjuvant efficacy despite being contradicted in other cancers;^449–452^ thus, various studies have been conducted to introduce targeted drugs into neoadjuvant therapy for CRC. For KRAS-wild-type patients with resectable colorectal liver metastases, although a few phase II trials stated potential efficacy for anti-EGFR agents,^453–455^ preoperative and postoperative use of cetuximab plus chemotherapy did not help and resulted in a shorter PFS (14.1 vs. 20.5 months, HR = 1.48, *p* = 0.03) than that seen with chemotherapy alone according to the EPOC trial.^456^ However, in another randomized controlled trial enrolling patients with KRAS-wild-type CRC with liver metastases, preoperative chemotherapy with cetuximab might have contributed to a better rate of R0 resection of the metastases (25.7% vs. 7.4%) and longer survival (3-year OS rate: 41% vs. 18%, *p* = 0.013; median survival time: 46.4 vs. 25.7 months, *p* = 0.013)^454^ over single-agent chemotherapy, suggesting that preoperative use of cetuximab is controversial for these patients. While perioperative administration of bevacizumab and chemotherapy for patients with liver metastatic CRC might be tolerable and helpful in prolonging survival, several phase II trials showed that recurrence was unavoidable, which lacks further validation.^457–460^

As for locally advanced rectal cancer, abundant phase II studies have been conducted for the neoadjuvant use of anti-EGFR or anti-VEGF therapy, with pooled estimated pCR values of 27% and 14% being reported for bevacizumab- and cetuximab-relevant regimens, respectively, via a meta-analysis of 32 previous studies.^448^ Although newer trials echoed the results from meta-analysis,^461^ to elucidate whether the targeted agents are truly effective in the neoadjuvant setting still requires larger population-based head-to-head, time-to-event data. It is worth noting that the efficacy of immune checkpoint modulators for the neoadjuvant treatment of CRC has been investigated in various ongoing trials, offering more potential choices in the future (NCT03926338, NCT03299660, NCT03102047, NCT04130854, NCT03854799, and NCT02754856).

### Interaction with the gut microbiota

The gut microbiota is closely related to carcinogenesis and tumor progression of CRC.^462–467^ Novel approaches for CRC screening and surveillance might be achieved through single or multiple microbial markers. Alteration of and intervention in the microbiome might also contribute to CRC treatment and further assist in predicting and monitoring treatment response and adverse events. Although most findings have been preliminary and await clinical validation, the gut microbiota represents one of most promising approaches for personized CRC therapy.

Current chemotherapy for CRC has been shown to potentially be mediated by the gut microbiota, which might respond to cytotoxic agents via alterations of their diversity, location, and metabolism.^466,468–470^ Specific types of organisms in the gut microbiota have been found to play a vital role in resistance to 5-FU and OX therapy by mediating autophagy.^471^ In addition, other gut residents might aggravate chemotherapy-related adverse reactions via microbial metabolism of chemotherapy agents such as IRI.^472^ There is limited evidence showing that the gut microbiota might interfere with targeted therapy; however, host–microbiome interactions in experimental settings might suggest some underlying clues for the microbial-mediated efficacy of targeted agents. Bile acids are among the major microbial metabolic products, which are also relevant to the initiation and progression of CRC.^473^ The crosstalk between the gut microbiota and the host intestinal cells is considered to be mediated by the progression of primary-secondary bile acid transformation, which mainly regulates the growth of colon epithelial cells via EGFR and nuclear farnesoid X receptor signaling.^474^ Thus, more investigation is needed to confirm that microbial interventions, such as the use of probiotics, could reduce intestinal inflammation through EGFR regulation. No further evidence has been presented stating that EGFR-targeted therapy might be interfered with by the gut microbiota. Similar conclusions can be drawn for anti-VEGF therapy, which may be even more related to the gut microbiota. An angiogenesis-mediated function for the gut microbiota was reported previously, by which the cancer microenvironment was formed and shaped.^475,476^ Probiotics might help to control local inflammation by downregulating the VEGF/VEGFR pathway in the liver and intestinal cells.^477,478^ Antibiotic use, which might dramatically reduce gut microbial diversity and density, was found to be related to poor survival in patients with metastatic CRC who had received bevacizumab therapy.^479^ However, the influence of the microbiota on anti-VEGF agents is still a matter of debate because of the controversial reported results^480,481^ in renal cell cancer-based studies stating an unclear role of antibiotics in VEGF-blockade therapy.

Interestingly, the gut microbiota turned out to be an indispensable factor for immune checkpoint blockade and affected responses and adverse reactions.^482–486^ Several strains of bacteria were related to the drug response,^487–492^ and further experiments showed that colonization by a combination of 11 strains of bacteria in germ-free mice might have enhanced the efficacy of immune checkpoint modulators, partially because the new bacterial infection led to stronger immune-protective infiltration and response from CD8+ T cells, as well as increased numbers of CD4+ T cells and CD103+/MHC class Ia-expressing dendritic cells.^493^ Antibiotic exposure was associated with reduced clinical activity in response to immunotherapy with a decreased PFS and OS in NSCLC (PFS: 1.9 vs. 3.8 months, HR = 1.5, *p* = 0.03; OS: 7.9 vs. 24.6 months, HR = 4.4, *p* < 0.01) and renal cancer (PFS: 1.9 vs. 7.4 months, HR = 3.1, *p* < 0.01; OS: 17.3 vs. 30.6 months, HR = 3.5, *p* = 0.03),^489^ findings that have been restated by recent studies that showed a dramatically reduced immunotherapeutic benefit (2 vs. 26 months, HR = 7.4) once antibiotics were given for several cancer types.^494,495^ Similarly, the efficacy of a PD-1 inhibitor in melanoma patients correlated with the gut microbiota, and reconstitution of the microbiota from PD-1 therapy-responding patients might result in an enhanced T cell response and improved PD-1-blocking effect, whereas transplantations from nonresponding patients had a negative outcome.^490–492^ Further metagenomics analysis identified *Akkermansia muciniphila* as the key bacteria that ameliorates PD-1 blockade, but this effect might be overcome by the administration of antibiotics.^496,497^ In addition, the gut microbiota might predict immunotherapy-related colitis because enriched levels of Firmicutes indicated a more frequent occurrence of ipilimumab-induced colitis.^488^ Currently, a few trials are in progress investigating whether immunotherapy can be modified by fecal microbiota transplantation (NCT04130763, NCT04116775, and NCT03341143). However, much remains to be learned about the gut microbiota in immune response regulation, and elucidating the underlying mechanism requires further investigation. Moreover, a lack of effective methods to precisely control the abundance or constitution of specific strains or groups of the gut microbiota limits the current opportunities for intervention.

## Other pathways

The development of new targeted agents based on pathways other than previously known ones appears to be rather slow. A few clinical trials concerning drugs aimed at targets such as IGF-1R, Wnt, Notch, Hedgehog, human death receptor 5 and TGF-β have been initiated, yet no attractive results have emerged so far. For example, the γ-secretase inhibitor RO4929097 in Notch blockade therapy and the Hedgehog pathway inhibitor vismodegib displayed little effect in phase II trials.^498,499^ The limited progress of anti-TGF-β and anti-Wnt therapy against CRC has also been reviewed.^500,501^ Agents such as COX-2 inhibitors were found to be helpful in CRC prevention in terms of Wnt inhibition; however, the development of other agents that might enhance chemotherapy sensitivity, yet direct CRC-control-targeted drugs with high affinity to single targets, still lags behind. The existence of crossover between these pathways has also made blockade therapy inefficient, and other obstacles, such as difficulties in selecting patients who will respond well, identifying outcome-monitor markers, and efficiently blocking specific targets, have appeared; however, these have not halted investigations into novel agents. Table 8 summarizes those agents under clinical investigation for various targets.

**Table 8:** Novel agents under clinical investigation

Name or ID	Targets	Condition	Phase	NCT identifier
WNT-974	Wnt	mCRC	Phase 1/2	NCT02278133
FOXY-5	Wnt	Multiple tumors including CRC	Phase 1	NCT02655952
		Multiple tumors including CRC	Phase 1	NCT02020291
		CRC	Phase 2	NCT03883802
LGK-974	Wnt	Multiple tumors including CRC	Phase 1	NCT01351103
RO4929097	Notch	CRC	Phase 1	NCT01198535
		CRC	Phase 2	NCT01116687
LY3039478	Notch	Solid tumors including CRC	Phase 1	NCT02784795
CB-103	Notch	Solid tumors including CRC	Phase 1/2	NCT03422679
MK-0752	Notch	Malignant neoplasms	Phase 1	NCT01243762
GEN-1029	DR5	Solid tumors including CRC	Phase 1/2	NCT03576131
INBRX-109	DR5	Solid tumors including CRC	Phase 1	NCT03715933
DS-8273a	DR5	CRC	Phase 1	NCT02991196
Conatumumab	DR5	mCRC	Phase 2	NCT00813605
		Solid tumors including CRC	Phase 2	NCT01327612
Vismodegib	Hedgehog	mCRC	Phase 2	NCT00636610
		Multiple tumors including CRC	Phase 2	NCT00959647
		First-line mCRC	Phase 1/2	NCT00625651
LDE225	Hedgehog	Solid tumors	Phase 1	NCT01576666
LY3200882	TGF	mCRC	Phase 1/2	NCT04031872
NIS-793	TGF	Multiple tumors including CRC	Phase 1	NCT02947165
KW-2450	IGF-1R	Advanced solid tumor	Phase 1	NCT00921336
IMC-A12	IGF-1R	mCRC	Phase 2	NCT00503685
Dalotuzumab	IGF-1R	mCRC	Phase 2	NCT00614393
		CRC	Phase 1	NCT00925015
Figitumumab	IGF-1R	Stage IV CRC	Phase 2	NCT00560560
Robatumumab	IGF-1R	Relapsed or recurrent CRC	Phase 2	NCT00551213
ganitumab	IGF-1R/HGF	KRAS-wild-type mCRC	Phase 1/2	NCT00788957
		mCRC	Phase 2	NCT00891930
		KRAS-mutant mCRC	Phase 2	NCT00813605

*CRC* colorectal cancer, *mCRC* metastatic colorectal cancer, *DR5* human death receptor 5, *TGF* transforming growth factor, *IGF-1R* insulin-like growth factor 1 receptor, *HGF* hepatocyte growth factor

## Future directions and conclusions

Human genomic, transcriptional, proteomic, and epigenetic details have never been as accessible as they have in the past few decades, owing to evolving sequencing technologies. Alterations in cell differentiation, proliferation, and survival resulting from genetic profile changes contribute to cancer initiation and development. On the basis of identifying these heterogeneities, treatments targeted to specific enzymes, growth factor receptors, and signal transducers make personalized cancer therapy possible, such that many oncogenic cellular processes can be efficiently interfered with, which holds the promise of precise cancer eradication and better patient care.

**Fig. 3:**
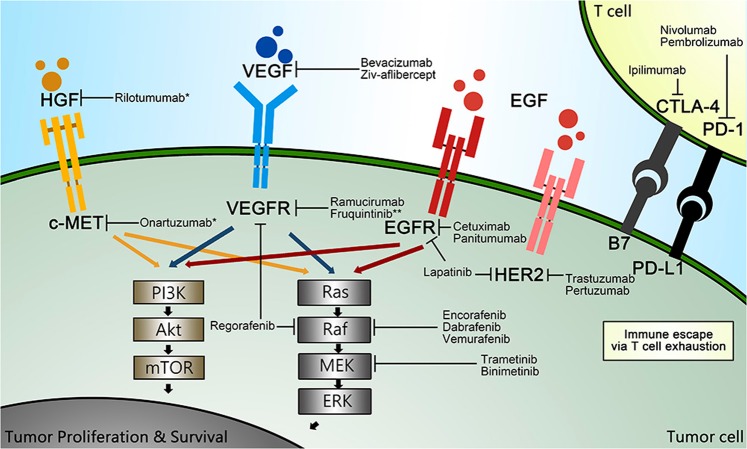
Overview of National Comprehensive Cancer Network (NCCN)-recommended targeted agents. HGF: hepatocyte growth factor; c-MET: mesenchymal–epithelial transition factor; VEGF: vascular endothelial growth factor; VEGFR: vascular endothelial growth factor receptor; EGFR: epidermal growth factor receptor; EGF: epidermal growth factor; HER2: human epidermal growth factor 2; CTLA-4: cytotoxic T lymphocyte-associated antigen 4; PD-1: programmed death-1; PD-L1: programmed death ligand 1; PI3K: phosphoinositide 3-kinase; AKT: protein kinase B, also known as PKB; mTOR: mammalian target of rapamycin; MEK: mitogen-activated protein kinase; ERK: extracellular signal-regulated kinase. *These agents have not been recommended by the NCCN. **This agent has been approved by the National Medical Products Administration of China (NMPA), but not by the United States of America Food and Drug Administration (FDA)

**Fig. 4:**
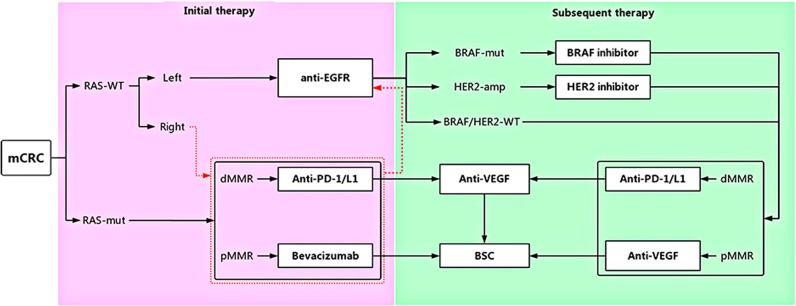
National Comprehensive Cancer Network (NCCN)-recommended strategy for metastatic colorectal cancer targeted therapy. mCRC: metastatic colorectal cancer; EGFR: epidermal growth factor receptor; VEGF: vascular endothelial growth factor; PD-1/L1: programmed death-1/programmed death ligand 1; dMMR: deficient mismatch repair; pMMR: proficient mismatch repair; HER2: human epidermal growth factor 2; BSC: best supportive care; WT: wild type; mut: mutated; amp: amplified. *The NCCN recommends initial administration of PD-1/PD-L1 therapy only in patients in poor functional status

After decades of development, great efforts have been devoted to updating CRC-targeted drugs for better patient compliance, fewer adverse events and more individualized treatment strategies. Current targeted agents for CRC and the NCCN-recommended strategy are summarized in Figs. 3 and 4. To date, there is no universal regimen that can easily treat every patient with equal efficacy, and our knowledge about CRC has also been advancing, resulting in the identification of novel targets.

CRC classification changes swiftly because of the rapidly developing pathological and immunological findings that might increase existing knowledge of cancer biological characteristics. The genetic heterogenicity of CRC has been identified, together with a comprehensive understanding of the different molecular pathways and genetic profiles involved. Updated classification of CRC led to the announcement of the Consensus Molecular Subtype (CMS) classification, which took both tumor pathological characteristics and gene expression into account:^502^ CMS1, enriched for inflammatory or immune genes; CMS2, canonical; CMS3, metabolic; and CMS4, mesenchymal. Although it is still in development, the CMS system might indicate the prognosis of CRC and help guide drug development and application.^503–505^ Data showed that the immune desert CMS2 and CMS3 subtypes responded preferably to anti-EGFR or anti-VEGF therapy, and patients in all four CMS classifications might have different mechanisms of immune evasion, which will enable tailored targeted therapy.^504,506,507^ There has been a changing trend from a clonal perspective to a clonal-stromal-immune perspective when multiple genetic profiles in genomics, transcriptomics, and immunity are considered before making decisions on comprehensive personized targeted therapy.^504^

**Fig. 5:**
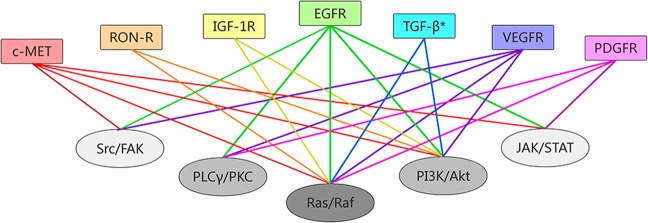
Crosstalk and bypass mechanisms between pathways. VEGFR: vascular endothelial growth factor receptor; EGFR: epidermal growth factor receptor; c-MET: mesenchymal–epithelial transition factor; IGF-1R: insulin-like growth factor 1 receptor; TGF-β: transforming growth factor-β; RON-R: recepteur d’Origine nantais; PDGFR: platelet-derived growth factor receptor; *with multiple isoforms

Although targeted therapy is associated with prolonged survival, there are several drawbacks: (1) The cost–benefit balance is questionable when current chemotherapy is much less expensive than extra targeted regimens, especially for those who may need multiple targeted agents. This is crucial because both producing targeted agents and testing necessary genetic markers remain costly, and the best result is longer survival and not full recovery. (2) Targeted therapy might cause extra adverse events. Level 3 or level 4 adverse events can be observed occasionally, and their incidence might increase when two or more targeted agents are combined. (3) Considering existing crossover and bypass mechanisms between pathways (Fig. 5), current drug resistance cannot be avoided, and acquired resistance adds further complications because the optimal solution might involve more treatment-related expense and toxicity. (4) Efficacy differs dramatically among people, leading to increased burdens associated with patient selection and surveillance. These issues may form a part of future directions for targeted drug development.

In general, we not only are encouraged by the fact that patients with CRC are living longer with plentiful choices of targeted treatments, of which one or more could ultimately be beneficial, but also expect even more individualized treatments to be developed that promote even longer survival, have fewer adverse reactions and have the potential for full recovery.
